# Diffusing protein binders to intrinsically disordered proteins

**DOI:** 10.1038/s41586-025-09248-9

**Published:** 2025-07-30

**Authors:** Caixuan Liu, Kejia Wu, Hojun Choi, Hannah L. Han, Xueli Zhang, Joseph L. Watson, Green Ahn, Jason Z. Zhang, Sara Shijo, Lydia L. Good, Charlotte M. Fischer, Asim K. Bera, Alex Kang, Evans Brackenbrough, Brian Coventry, Derrick R. Hick, Seema Qamar, Xinting Li, Justin Decarreau, Stacey R. Gerben, Wei Yang, Inna Goreshnik, Dionne Vafeados, Xinru Wang, Mila Lamb, Analisa Murray, Sebastian Kenny, Magnus S. Bauer, Andrew N. Hoofnagle, Ping Zhu, Tuomas P. J. Knowles, David Baker

**Affiliations:** 1 Department of Biochemistry, University of Washington, Seattle, WA USA; 2 Institute for Protein Design, University of Washington, Seattle, WA USA; 3 Biological Physics, Structure and Design Graduate Program, University of Washington, Seattle, WA USA; 4 Key Laboratory of Epigenetic Regulation and Intervention, Institute of Biophysics, Chinese Academy of Sciences, Beijing, China; 5 University of Chinese Academy of Sciences, Beijing, China; 6 Department of Laboratory Medicine and Pathology, University of Washington, Seattle, WA USA; 7 Centre for Misfolding Diseases, Yusuf Hamied Department of Chemistry, University of Cambridge, Cambridge, UK; 8 Laboratory of Chemical Physics, National Institute of Diabetes and Digestive and Kidney Diseases, National Institutes of Health, Bethesda, MD USA; 9 Cavendish Laboratory, Department of Physics, University of Cambridge, Cambridge, UK; 10 Howard Hughes Medical Institute, University of Washington, Seattle, WA USA

**Keywords:** Protein design, Protein aggregation, Diagnostic markers

## Abstract

Proteins that bind to intrinsically disordered proteins (IDPs) and intrinsically disordered regions (IDRs) with high affinity and specificity could be useful for therapeutic and diagnostic applications^1–4^. However, a general methodology for targeting IDPs or IDRs has yet to be developed. Here we show that starting only from the target sequence of the input, and freely sampling both target and binding protein conformations, RFdiffusion^5^ can generate binders to IDPs and IDRs in a wide range of conformations. We used this approach to generate binders to the IDPs amylin, C-peptide, VP48 and BRCA1_ARATH in diverse conformations with a dissociation constant (*K*_d_) ranging from 3 to 100 nM. For the IDRs G3BP1, common cytokine receptor γ-chain (IL-2RG) and prion protein, we diffused binders to β-strand conformations of the targets, obtaining *K*_d_ between 10 and 100 nM. Fluorescence imaging experiments show that the binders bind to their respective targets in cells. The G3BP1 binder disrupts stress granule formation in cells, and the amylin binder inhibits amyloid fibril formation and dissociates existing fibres, enables targeting of both monomeric and fibrillar amylin to lysosomes, and increases the sensitivity of mass spectrometry-based amylin detection. Our approach should be useful for creating binders to flexible IDPs or IDRs spanning a wide range of intrinsic conformational preferences.

## Abstract (web summary)

Using RFdiffusion, a general method for targeting intrinsically disordered proteins and regions for protein design has been developed.

## Main

IDPs and structured proteins with intrinsically disordered regions (IDPRs) are abundant in nature, representing approximately 60% of the human proteome^6^. They carry out key biological functions without adopting a single well-defined structure. Many of these proteins are well-established biomarkers in clinical care and biomedical research. IDPs and IDPRs adopt a continuum of states with full or segmental disorder^7^; this structural plasticity allows IDPs and IDPRs to adapt to different partners and conditions. Designed binders specific for IDPs or IDPRs could be valuable for clinical diagnosis, therapeutic development and scientific research^1–4^, but the lack of a single well-defined state makes them challenging targets. Current methods largely rely on antibodies, which are limited by high production costs, reproducibility and complex engineering requirements^8,9^; the dynamic nature of disordered proteins can also complicate the elicitation of antibodies as the targets can be rapidly degraded following immunization. Computational protein design has created binders of peptides in extended β-strand^10,11^, helical^12^ and polyproline II conformations^13^. Although powerful, these methods require pre-specification of the target peptide geometry, which can be limiting because the optimal conformation given the intrinsic sequence biases of the peptide, and the opportunities for making high-affinity interactions, may be quite irregular. Collectively, these approaches have focused primarily on relatively short, structured peptides.

**Fig. 1:**
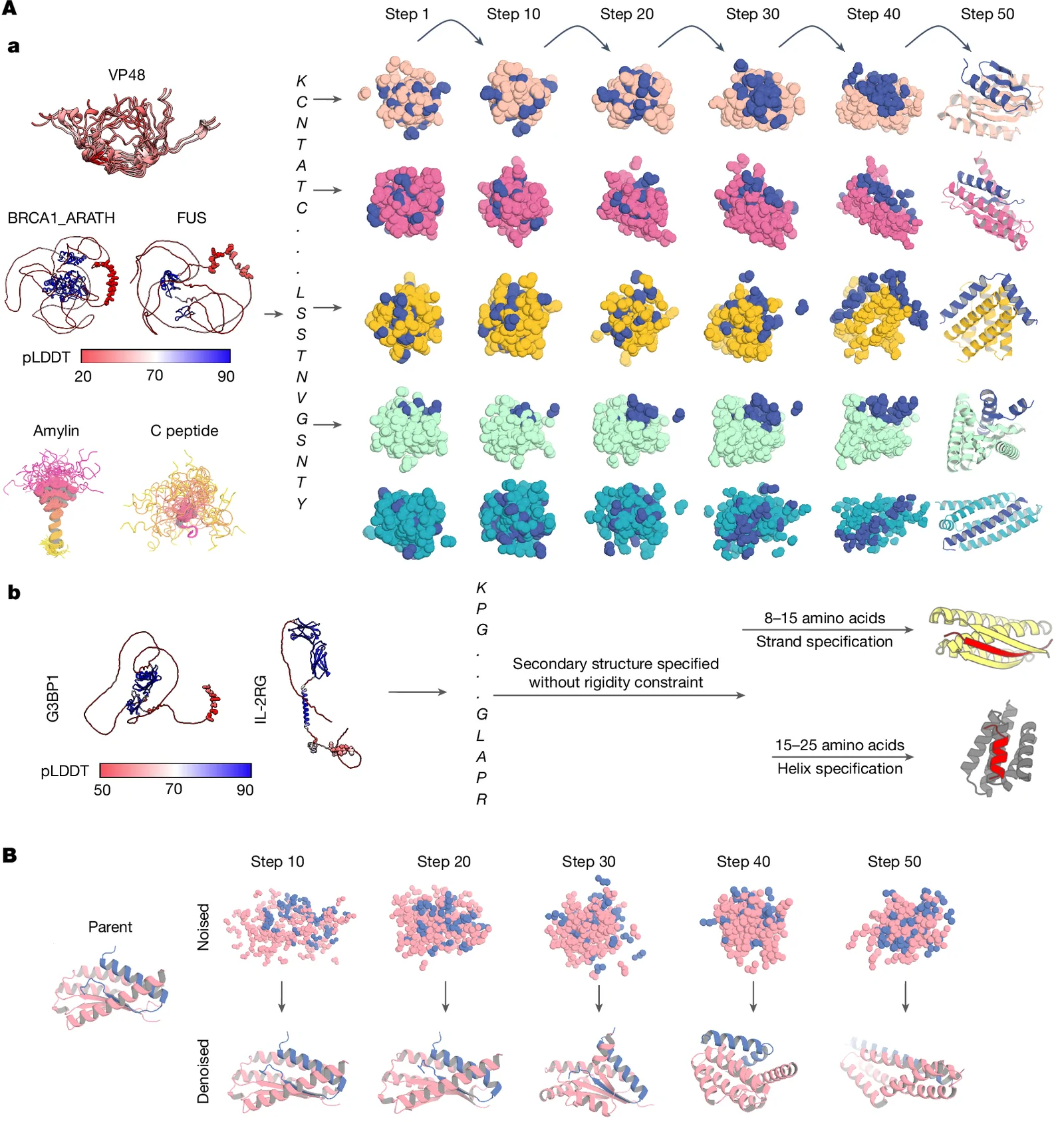
Design strategies for binding conformational flexible IDPs or IDRs. **A**, Design approach. **Aa**, Disordered structures of the selected targets. Left, AF2 structure predictions for VP48, BRCA1, ARATH and FUS, coloured by pLDDT scores^25^, and NMR structures of amylin (PDB ID: 2KB8) and CP (PDB ID: 1T0C). Targeted regions are shown as spheres. Right, diffusion models for proteins are trained to recover noised protein structures and to generate new structures by reversing the corruption process through iterative denoising of initially random noise into a realistic structure. Five representative trajectories are shown for amylin, which result in five different conformations of the target. **Ab**, Diffusion with constrained secondary structure. Left, AF2 predictions for G3BP1 and IL-2RG^25^. Right, a modified version of RFdiffusion was trained, allowing for specification of the secondary structure of a region to be helix or strand conformations, along with the sequence. **B**, Two-sided partial diffusion. Varying the extent of initial noising (top row) enables control over the extent of the introduced structural variation (colours indicate new designs, and grey denotes the parent design).

We sought to develop a general approach to design high-affinity binders for IDPs that starts from the target sequence alone, does not require pre-specification of the target geometry and is applicable to IDPs of varying lengths (Fig. 1Aa). We reasoned that a version of RFdiffusion trained on two chain systems from the Protein Data Bank (PDB), noising the structure on one and providing only the sequence for the second, could have such a capability. To target shorter IDRs, we reasoned that strand pairing, as used by Sahtoe et al. using Rosetta^11^, coupled with RFdiffusion^5^ to sample the many different possible variations of strand conformation, could provide a general approach to maximizing interactions over a short region as backbone–backbone hydrogen bonds contribute to binding energy in addition to sidechain–sidechain interactions (Fig. 1Ab). For testing this approach, we selected a wide diversity of disordered IDPs and protein targets with lengths ranging from 31 to 941 residues, and targeted regions within these proteins that range from having some residual secondary structure propensity to having essentially no secondary structure propensity (as assessed with the disorder prediction programs IUpred3 (refs. ^14,15^) and Jpred4 (ref. ^16^)); we evaluated binding of the designs to the full-length targets using a combination of biochemical assays and in cell fluorescence imaging. During the diffusion calculations, both the target and the designed binder populate a wide range of conformations, and shape matching structures for both emerge that enable extensive interactions between the two. The resulting designed binders are well-folded protein that interact with a specific subregion of the target in a specific conformation rather than with the full disordered ensemble; such an induced fit mechanism for binding, where the binder selects a specific conformation out of a broad ensemble, is widespread in interactions between folded proteins and IDRs in nature^17^.

RFdiffusion was previously used to generate binders to bioactive peptide hormones restricted to helical conformations^12^; here we began by investigating the application of the approach to IDPs in a much broader range of conformations (the sequences of many targets are not compatible with uninterrupted helical conformations). We first experimented with designing binders to the human islet amyloid polypeptide (hIAPP; also known as amylin), a 37-residue hormone co-secreted with insulin by pancreatic islet β-cells to modulate glucose levels^18,19^. Cysteine residues 2 and 7 form a disulfide bridge that is critical for the full biological activity of amylin^18^. NMR studies conducted in lipid environments or under SDS micelle binding conditions have indicated helical propensity in amylin fragments^20,21^; the overall structure appears to be intrinsically disordered^22,23^.

We used flexible target fine-tuned RFdiffusion to design binders against amylin using only sequence as input: the structure of the binding protein, the amylin conformation and the binding mode are entirely unspecified. Starting from the amino acid sequence of amylin, RFdiffusion generated complexes spanning a wide range of conformations for both amylin and the designed binders. Representative design trajectories are shown in Supplementary Video 1; starting from a random distribution of residues of both amylin and binder, in sequential denoising steps, the amylin adopts different conformations, whereas the binder residue distribution shifts to surround amylin and progressively organizes into a folded structure that cradles nearly the entire surface of the target structure (Fig. 1Aa). Sequences were designed for the generated backbones using ProteinMPNN^24^, and filtered using AlphaFold2 (AF2)^25^ for the monomer conformation and AF2 initial guess for the complex^26^.

**Fig. 2:**
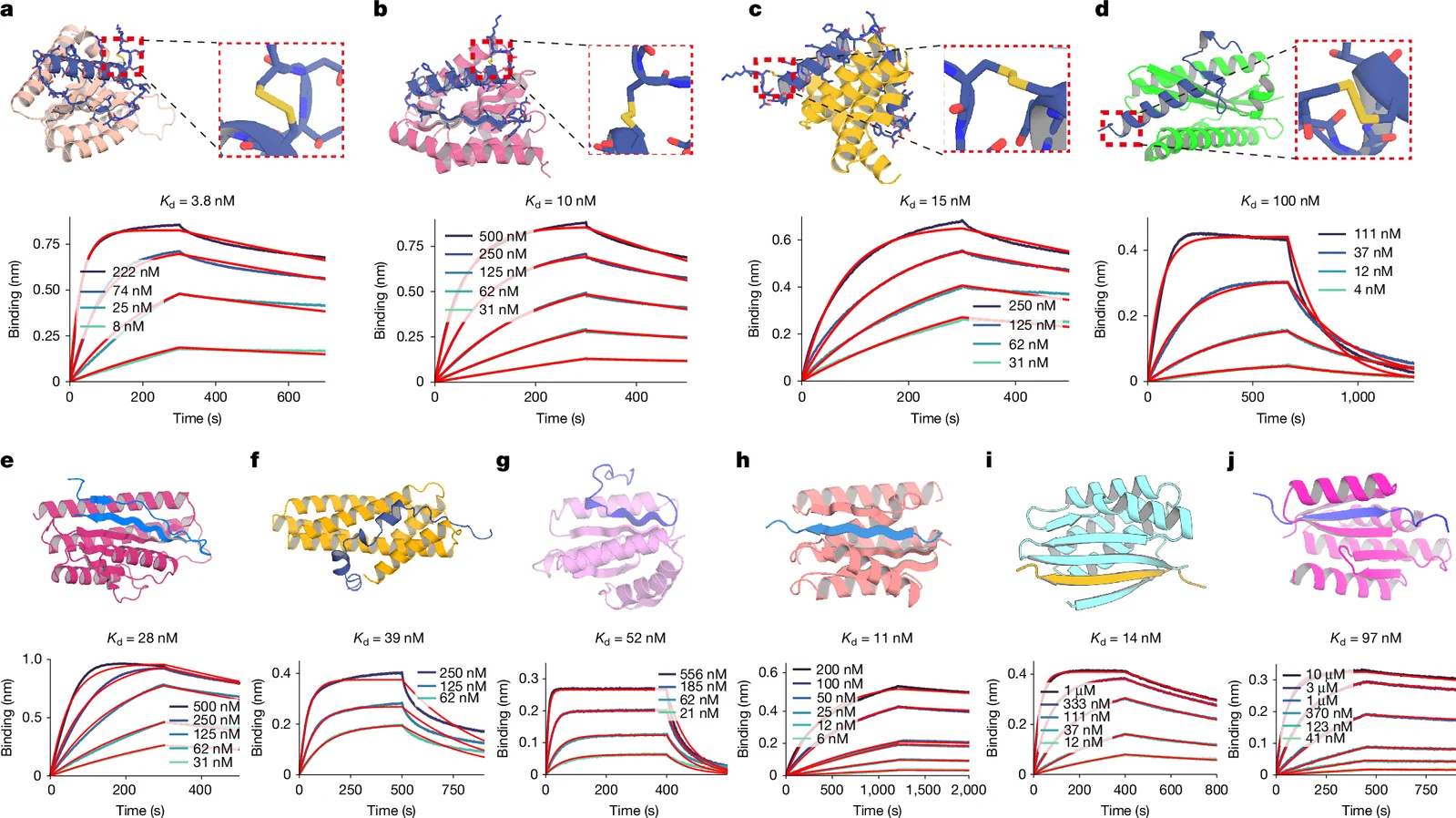
Design of intrinsically disordered region binders. **a**–**d**, Computational design models of amylin and designed binders amylin-68n_αβ_ (**a**), amylin-36_αβ_ (**b**), amylin-75_αα_ (**c**) and amylin-22_αβL_ (**d**) generated using sequence input diffusion (top), with a zoom-in view of disulfide bonds formed during diffusion. The secondary structure of amylin is indicated by the binder name subscripts. BLI measurements of the designed binder–amylin interaction are shown below the design models. **e**–**g**, Design of binders for CP (**e**), VP48 (**f**) and BRCA1_ARATH (**g**) using sequence input diffusion. **h**–**j**, Design of binders for G3BP1 (**h**), prion protein (**i**) and IL-2RG (**j**) with strand specification during diffusion.

**Extended Data Fig. 1:**
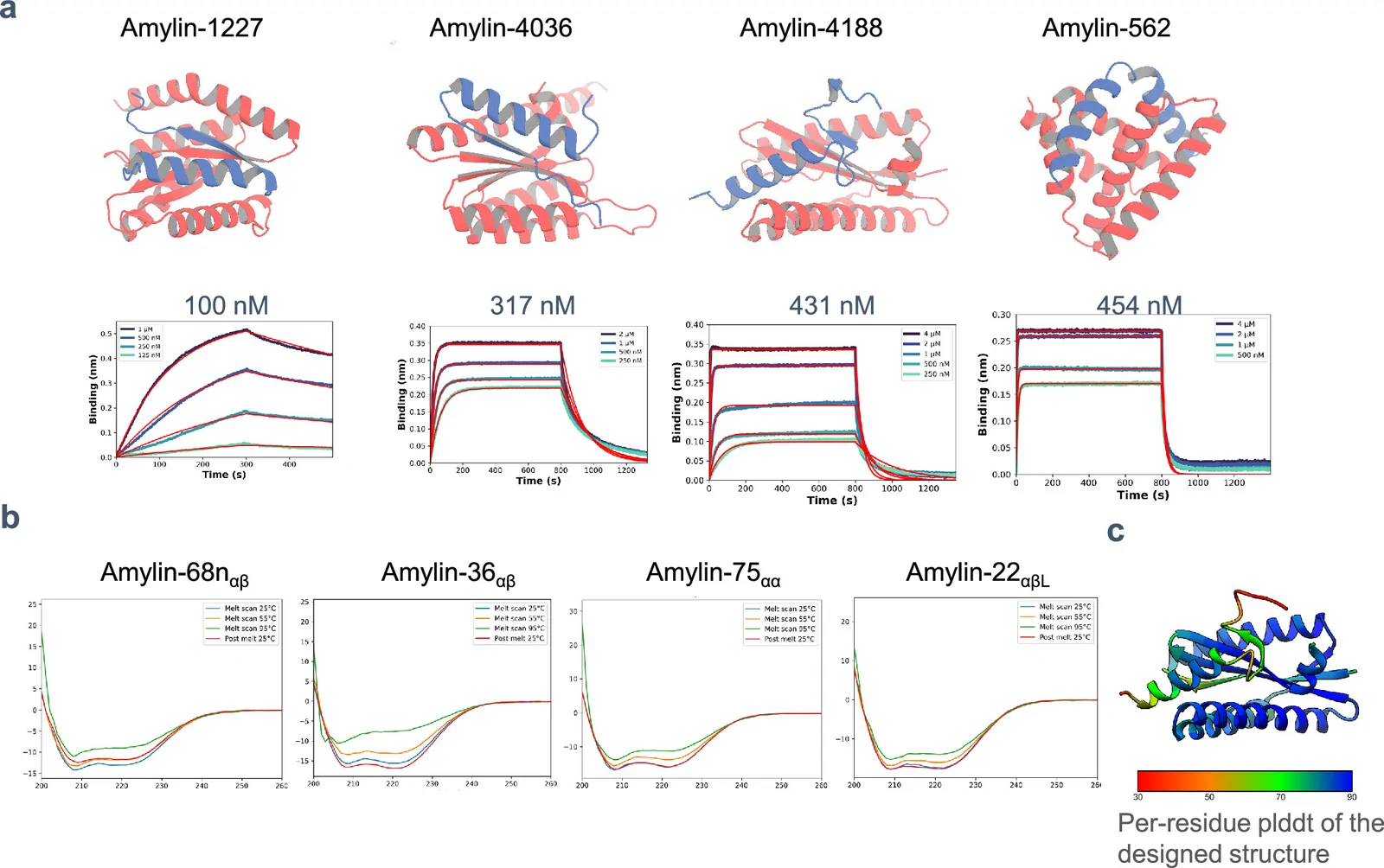
Diffusing de novo binder design to amylin. **a**, Top, the designed structures of four initial hits, Amylin-1227, −4036, −4188, −562, which serve as starting points for two-sided partial diffusion. Bottom, bio-layer interferometry (BLI) result of the four hits revealing the binding affinity of the four initial hits are 100, 317, 431, 454 nM, respectively. **b**, Circular dichroism data show that the four optimized binders have helical secondary structure and are stable up to 95 °C (inset). **c**, The per residue pLDDT (predicted Local Distance Difference Test) plotting of Amylin- Amylin-22_αβL_ complex in design.

**Extended Data Fig. 2:**
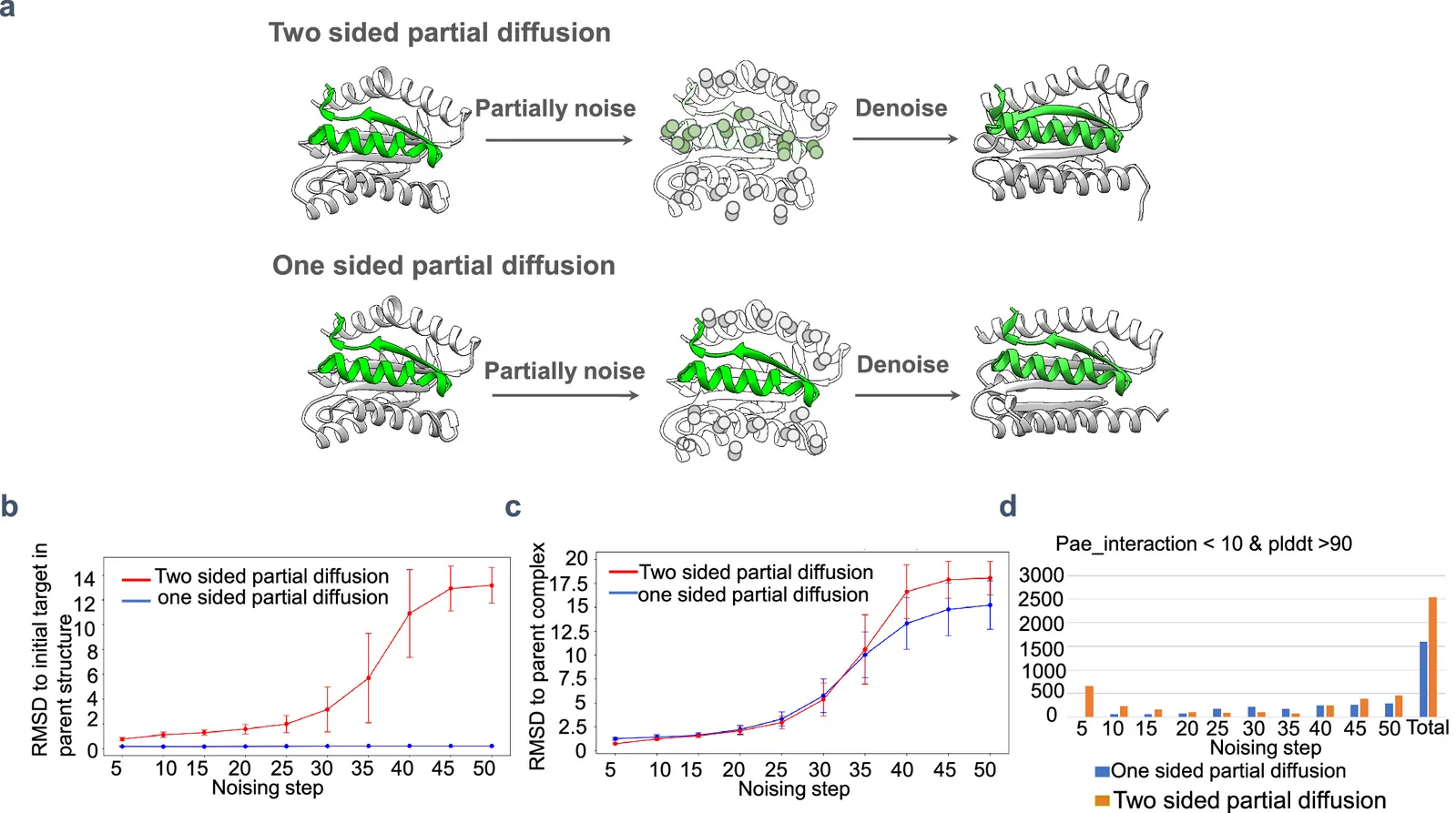
Two sided partial diffusion and comparison with one sided partial diffusion. **a**, Top, two-sided partial diffusion allows simultaneous conformational changes in both the target and the binder. Bottom, one-sided partial diffusion solely diversifies the conformation of the binder while keeping the target fixed. **b**, Two-sided partial diffusion (in red) diversifies the target while one sided partial diffusion (in blue) keeps the target fixed. **c**, The target-binder complex diverse magnitudes of two-sided (in red) and one-sided partial diffusion (in blue) remain comparable before nosing step 35, after step 35, the diverse magnitude of two-sided partial diffusion is larger than one sided one. **d**, Take the interface pAE_interaction <10, pLDDT >90 as cutoff criterion, two-sided partial diffusion yielded designs with generally better metrics than one sided diffusion. At steps 25, 30, and 35 exclusively, one-sided partial diffusion exhibited superior performance. However, in practical cases, we typically operate within fewer than 25 steps to remain the main features of parent structure.

We tested 96 designs against amylin in various non-helical conformations (as diffusion generated binders to helical peptides have previously been described), and found that these had binding affinities ranging from 100 nM to 454 nM (Extended Data Fig. 1a). To optimize binding affinity further, we implemented a two-sided partial diffusion approach (see Methods; in contrast to one-sided partial diffusion, which diversifies the binder conformation but keeps the target fixed, two-sided partial diffusion samples varied target and binder conformations (Fig. 1B and Extended Data Fig. 2a)). We found that two-sided diffusion yielded designs with better metrics than one-sided diffusion, probably because the target conformation can adapt to that of binder, resulting in greater shape complementarity and more extensive interactions (Extended Data Fig. 2). Among the 174 top-ranked designs spanning αβ, αβL and αα amylin conformations, 107 bound amylin; the highest affinity binders (amylin-68n_αβ_, amylin-36_αβ_, amylin-75_αα_ and amylin-22_αβL_), which bind amylin in different conformations, have affinities of 3.8 nM, 10 nM, 15 nM and 100 nM, respectively (Fig. 2a–d). Although the amylin adopts very different conformations in different designs, the diffusion process was able to maintain the disulfide bond, key to amylin function, in all designs^18^ (Fig. 2a–d). Circular dichroism studies showed that all four binders were largely helical as designed and thermostable up to 95 °C (Extended Data Fig. 1b).

**Extended Data Fig. 3:**
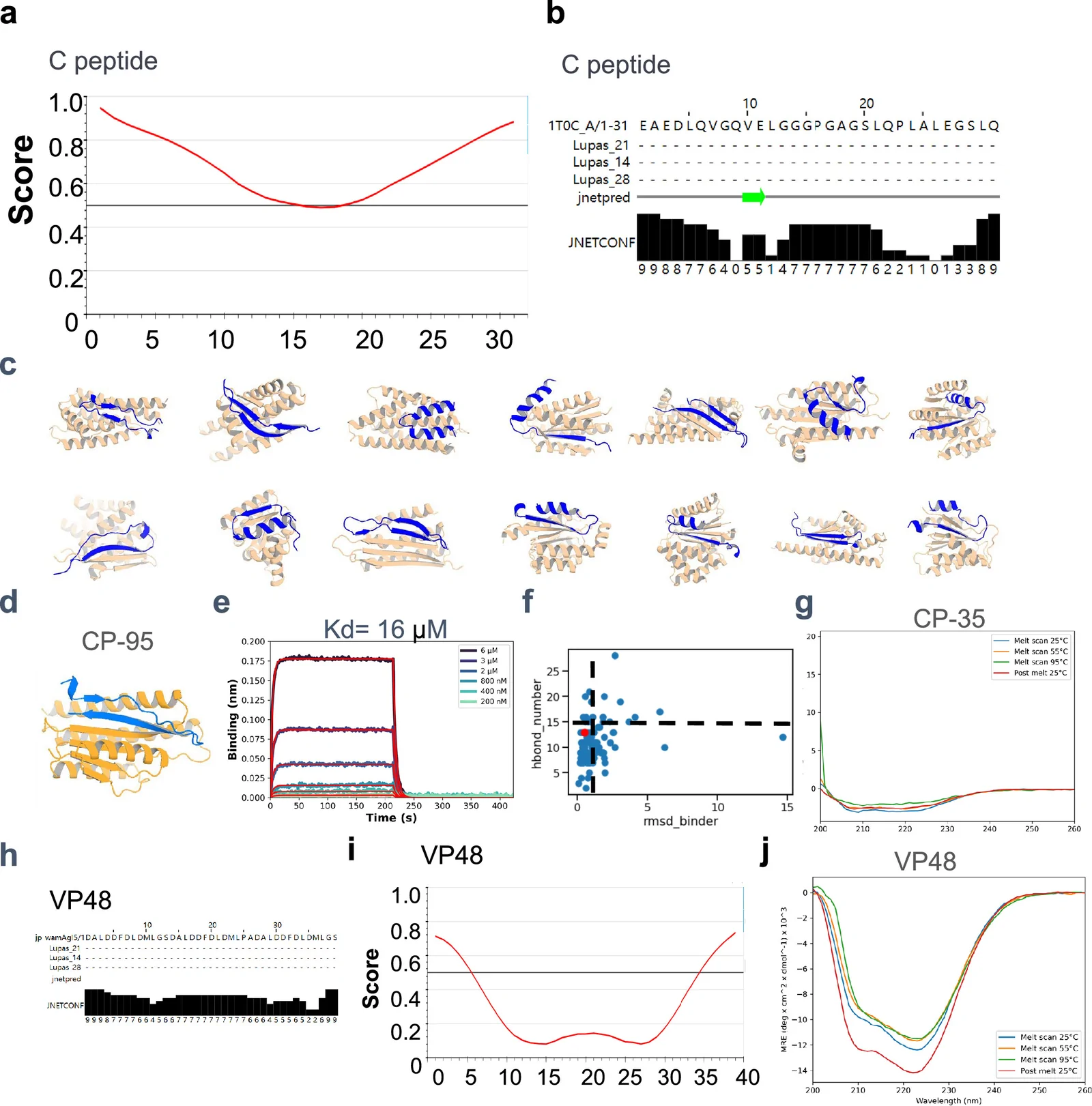
Diffusing de novo binder design to CP and disorder and secondary structure prediction for CP and VP48. **a**, IUPred3 predictions for the CP, the predicted disorder scores remain above 0.5 across the targeted regions, indicating that CP is intrinsically disordered^14,15^. **b**, JPred4 (ref. ^16^) secondary structure predictions for CP, three residues within the target region show strand propensity (green arrows). **c**, Sequence-input diffusion was carried out, allowing CP to sample diverse conformations. Representative examples are shown here. The diverse conformations of CP and protein binder are rendered in blue and wheat colour, respectively. **d**, Design model of the initial hit CP-95 which was also the starting point of two-sided partial diffusion. **e**, The BLI data revealed that the binding affinity of the initial hit CP-95 is 16 μm. **f**, Scatter plot showing the distribution of designs based on the number of hydrogen bonds (hbond_number) and the RMSD of the binder (rmsd_binder). Each blue dot represents a design, while the red dot marks a validated hit. The dashed black lines indicate the cutoff values based on the initial hit criteria (hbond_number = 13 and rmsd_binder = 0.545). **g** and **j**, Circular dichroism data show that the binder CP-35 (**g**) and VP48-2 (**j**) have helical secondary structure and are stable up to 95 °C (inset). **h**, Jpred4 predictions for VP48, indicating VP48 doesn’t have secondary structure propensity. **i**, Predicted disorder profiles for VP48 generated by IUPred3 indicate low disorder propensity in its central segments. Combined with the low pLDDT scores predicted by AlphaFold (Fig. 1A), these results suggest that VP48 is structurally ambiguous.

C peptide (CP; 31 residues) is secreted by islet β-cells as part of proinsulin^27^; measurement of plasma CP levels is used in the diagnosis of type I and type II diabetes^28^. The NMR structure of CP shows that it is disordered and highly dynamic^29^ (Fig. 1A), consistent with predictions from IUpred3 (refs. ^14,15^) and Jpred4 (ref. ^16^) (Extended Data Fig. 3a,b). Of 96 designs generated using sequence-input diffusion (Extended Data Fig. 3c), one in which the CP formed a long strand, followed by a long dynamic loop and a small strand paired with the long strand, had weak binding affinity (Extended Data Fig. 3d,e). Guided by the observation that this design had more hydrogen bonds between target and binder (13) than all but 5 of the 96 designs (Extended Data Fig. 3f), we again used two-sided partial diffusion and selected designs with larger numbers of hydrogen bonds. Six out of 95 designs bind to CP with better than 100 nM binding affinity; the highest affinity binder (CP-35) had a *K*_d_ of 28 nM (Fig. 2e) and was thermostable up to 95 °C (Extended Data Fig. 3g).

We next targeted VP48 (39 residues), a potent activator of transcription^30^ that Jpred4, IUPred3 and AF2 suggest is flexible and lacks defined secondary structure (Fig. 1A and Extended Data Fig. 3h,i). Of the 95 designs tested, the best binder with an affinity of 750 nM recognizes VP48 in a conformation with three short helical fragments connected by long loops. Further partial diffusion optimization yielded a design with a *K*_d_ of 39 nM (Fig. 2f) that was thermostable up to 95 °C (Extended Data Fig. 3j).

**Extended Data Fig. 4:**
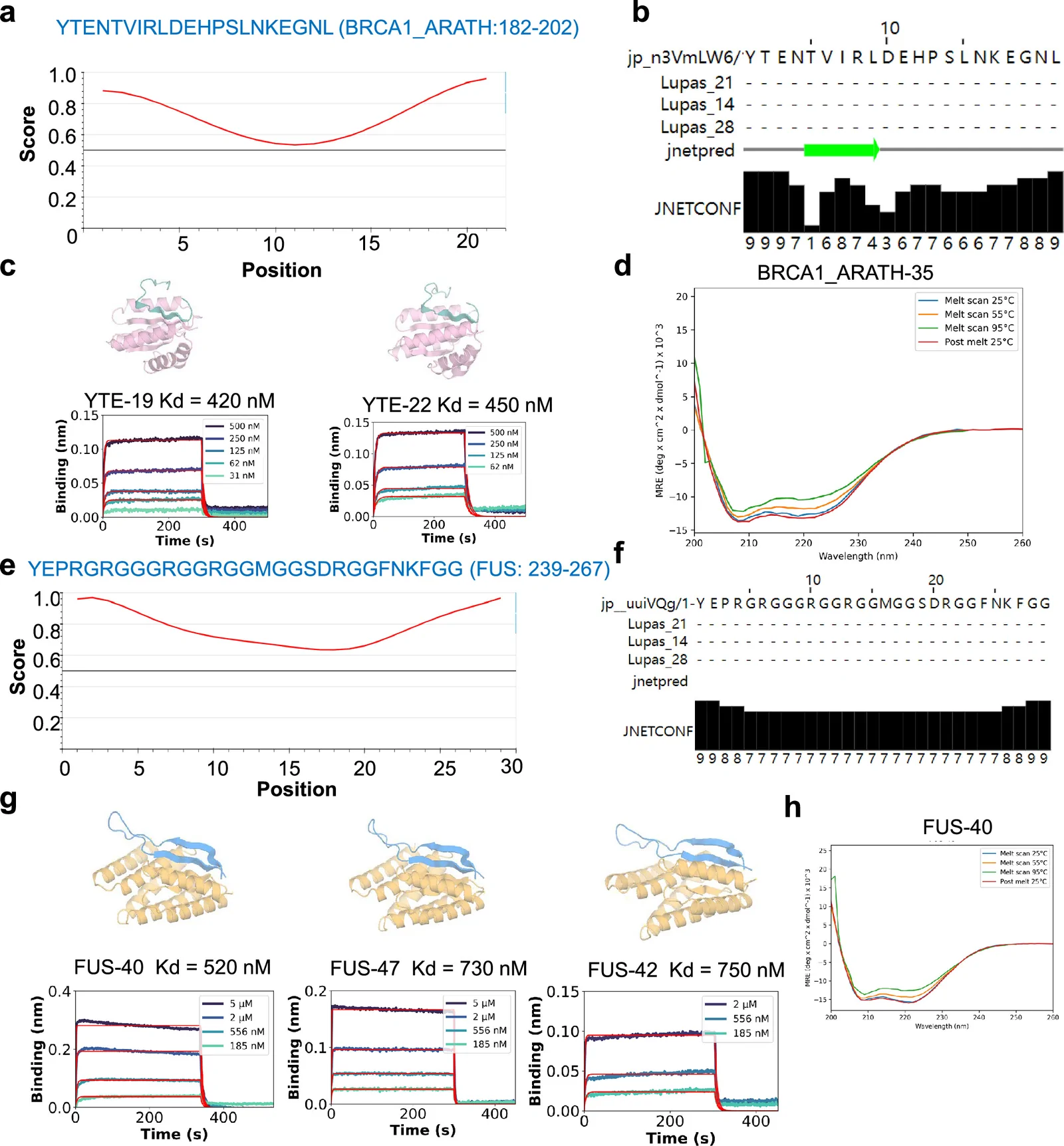
Diffusing de novo binder design to BRCA1_ARATH and FUS. **a**,**e**, IUPred3 predictions for the targeted regions on BRCA1_ARATH (**a**) and FUS (**e**); the predicted disorder scores remain above 0.5 across the targeted regions, indicating that they are intrinsically disordered. **b**, JPred4 secondary structure predictions for the targeted regions on BRCA1_ARATH; five residues within the target region show strand propensity (green arrows). **c**, Top, the designed structures of two initial hits of BRCA1_ARATH, YTE-19 and YTE-22, which serve as starting point of two-sided partial diffusion. Bottom, the BLI result of the two hits, revealing that the binding affinities of the two initial hits are 420 and 450 nM, respectively. **d**,**h**, Circular dichroism data show that the binder BRCA1_ARATH-35 (**d**) and FUS-40 (**h**) have helical secondary structure and are stable up to 95 °C (inset). **f**, No secondary structure is predicted within the targeted region for the targeted region on FUS predicted by Jpred4. **g**, Top, the designed structures of three hits of FUS, FUS-40, FUS-42 and FUS-47. Bottom, the BLI result reveals that the binding affinity of the three initial hits are 520, 730 and 750 nM, respectively.

To assess whether our method can target long IDPs, we chose BRCA1_ARATH (941 residues), a plant homologue of human BRCA1 involved in DNA repair in plants^31^; binders of the IDRs of BRCA1_ARATH could help to dissect its molecular function in DNA damage response and genome maintenance. We targeted a 21-residue sequence, YTENTVIRLDEHPSLNKEGNL (position 182–202), predicted to be largely disordered (Fig. 1A and Extended Data Fig. 4a,b). Using sequence input diffusion, 2 of 41 tested designs showed binding at approximately 450 nM by biolayer interferometry (BLI; Extended Data Fig. 4c). Two-sided partial diffusion optimization yielded a design with a *K*_d_ of 52 nM (Fig. 2g), which was thermostable up to 95 °C (Extended Data Fig. 4d).

To further evaluate whether our method can target highly dynamic and hydrophilic IDRs, we selected FUS (526 residues), a DNA/RNA-binding protein involved in various cellular processes including transcription regulation, RNA splicing, RNA transport and DNA repair^32^. We focused on a 29-residue segment, YEPRGRGGGRGGRGGMGGSDRGGFNKFGG (positions 239–267), predicted to be disordered (Fig. 1A and Extended Data Fig. 4e,f) with 52% glycine, serine content and charged or polar residues. Of 94 designs tested, 3 showed binding, with the top design having an affinity of 520 nM and thermostability up to 95 °C (Extended Data Fig. 4g,h).

## Targeting short IDRs via strand binding

Consistent with previous observations using the non-deep learning Rosetta method^11^, we found that for targeting shorter segments, the RFdiffusion-generated designs with the best metrics often made extensive β-strand interactions to targets adopting β-strand conformations. To increase the efficiency of generating such designs, we incorporated into the RFdiffusion sequence input approach the ability to define the secondary structure of the target (see Methods), to enable the specification of either the entire or a portion of the target sequence in helical, strand or loop conformation. This is particularly important for strand conformations, which can vary considerably in actual 3D coordinates; the coordinate specifying approach used by Vasquez et al.^12^ for helical peptides would be less efficient for targeting strands as many designs would have to be carried out for β-strand conformations with different twists and other structural variations. We found that strand-based interfaces can arise when the target sequence lacks intrinsic strand propensity, and even with overall strand prespecification, the β-strands have considerable flexibility to conform to the designed binder via induced fit. To explore the power of this approach, we used it to design binders to three IDR-containing targets.

**Extended Data Fig. 5:**
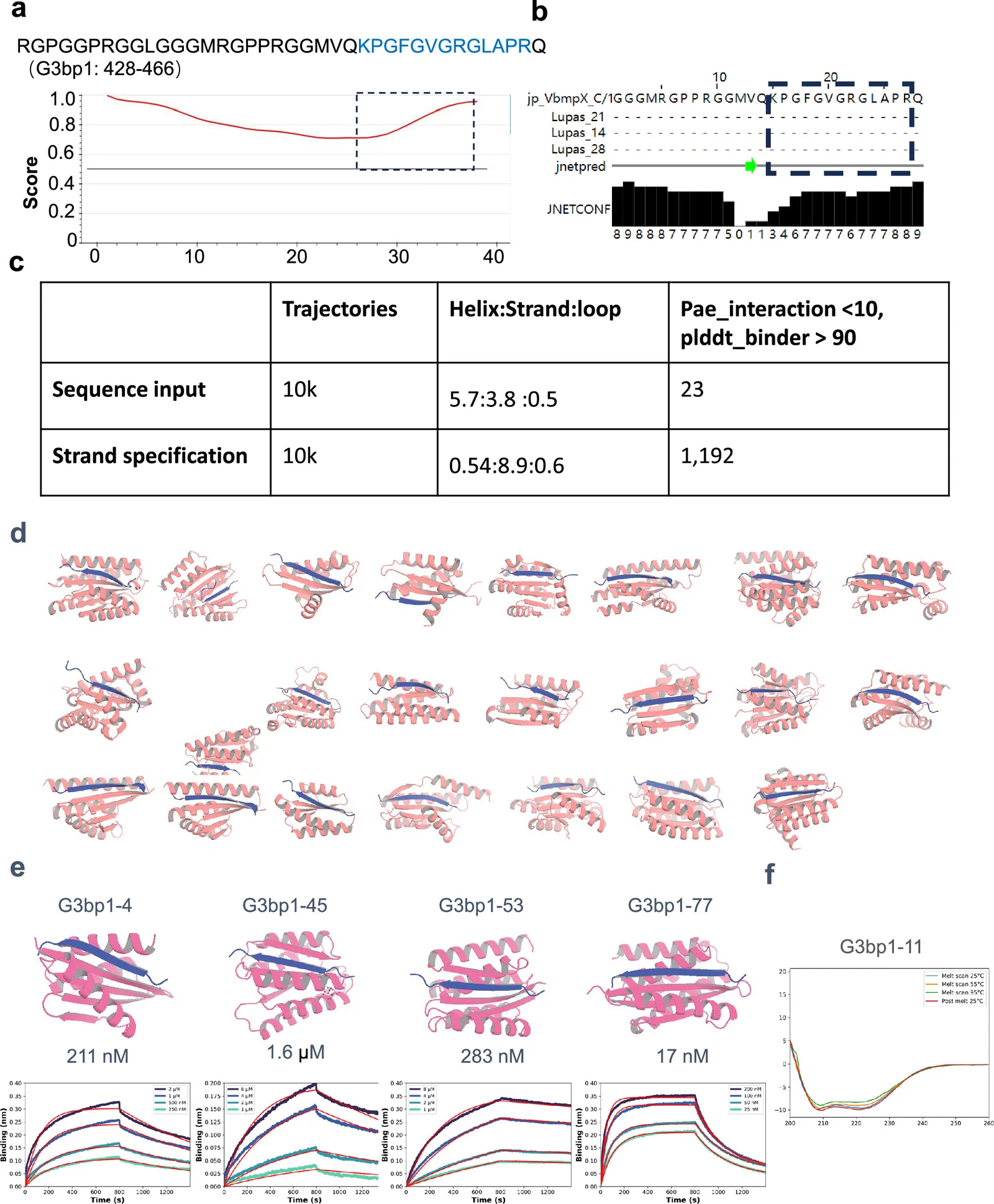
Diffusing de novo binder design to G3BP1^RBD^. **a**, IUPred3 predictions for the C-terminus of G3BP1, with the targeted regions highlighted by black dashed boxes and the corresponding sequences shown in blue, indicating that the segment we targeted on G3bp1 is intrinsically disordered. **b**, JPred4 secondary structure predictions for the same regions on G3BP1; no secondary structure is predicted within the targeted region. **c**, Comparative analysis of structural outcomes between sequence input and strand specification approaches in protein design. The table presents the number of designs (10k) and the distribution of secondary structures (helix:strand:loop) for both methods. This table counts the successful cases where the pAE_interaction is less than 10 and the plddt_binder score is greater than 90, noting 23 successes with sequence input and 1,192 with strand specification. This reflects an approximately 51-fold increase in efficacy with the strand specification method, highlighting its superior performance in achieving desired structural configurations. **d**, The 23 successful cases designed using sequence input RFdiffusion all feature targets in strand conformation. **e**, Design models (top) and BLI data (bottom) of the four initial hits of G3BP1^RBD^ which was also the starting point of two-sided partial diffusion. **f**, Circular dichroism data show that the G3bp1-11 binder has helical secondary structure and is stable up to 95 °C (inset).

G3BP1 (466 residues) is a central node within the core stress granule network^33^ and has a crucial role in RNA metabolism and stress response, with a disordered RNA-binding domain (RBD; Fig. 1A and Extended Data Fig. 5a,b) mediating interactions with RNA molecules, regulating RNA metabolism and contributing to the assembly and disassembly of stress granules. RFdiffusion with sequence-only specification of the RBD domain of G3BP1 (G3BP1^RBD^; Supplementary Table 1) yielded designs with the targeted region adopting a roughly 5.7:3.8:0.5 ratio for helix:strand:loop (Extended Data Fig. 5c), but only the 23 strand-containing designs had AF2 predicted aligned error (pAE) interaction < 10 and predicted local distance difference test (pLDDT) binder > 90 (Extended Data Fig. 5d). On the basis of these observations, we specified the secondary structure as a strand which increased the fraction of designs passing the filters more than 50-fold. Five out of 78 designs bound to G3BP1^RBD^, with the tightest having a binding affinity at 17 nM. Following two-sided partial diffusion (Extended Data Fig. 5e), the tightest binder (G3BP1-11) had an affinity of 11 nM (Fig. 2h) and was thermostable up to 95 °C (Extended Data Fig. 5f).

**Extended Data Fig. 6:**
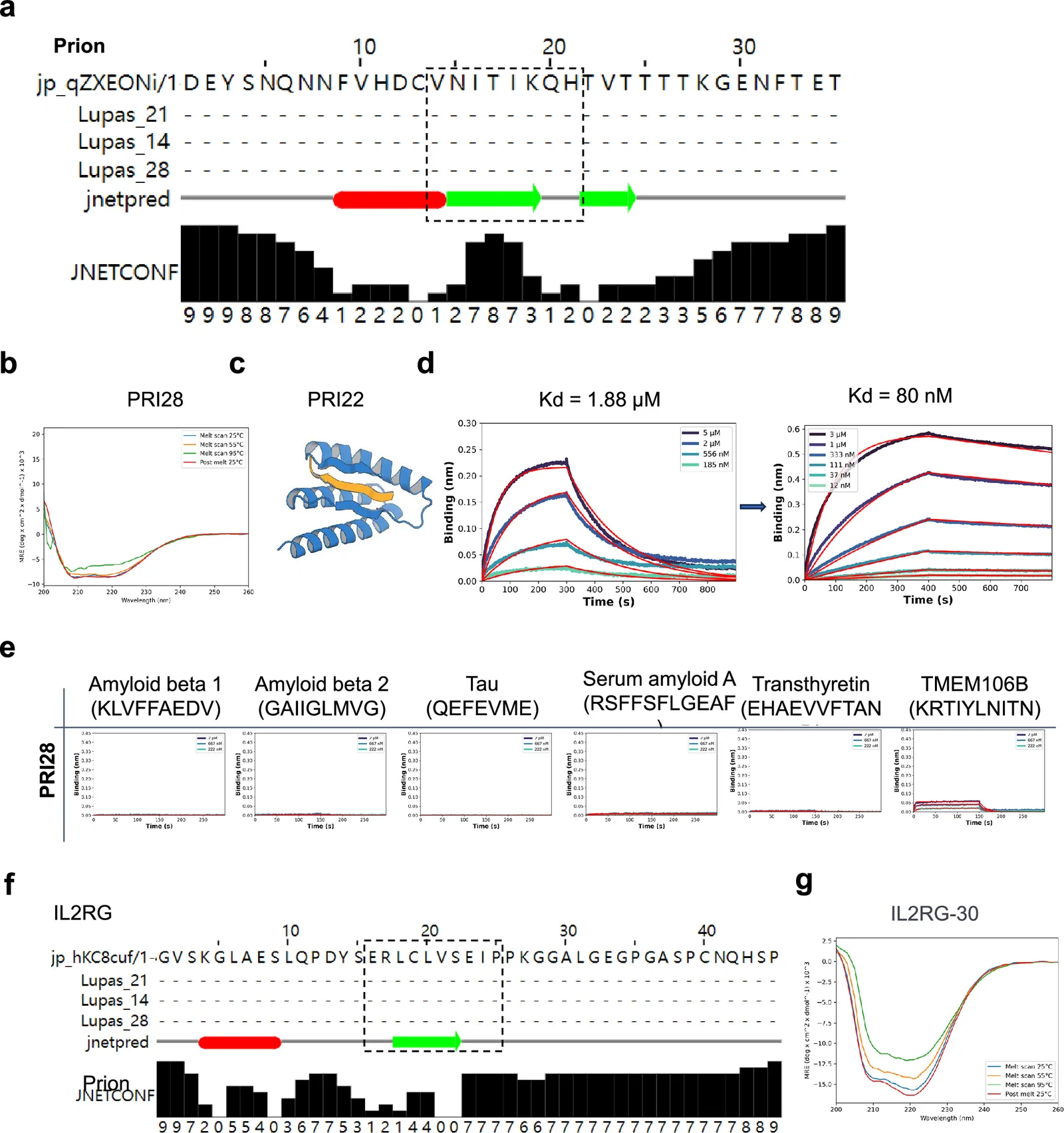
Diffusing de novo binder design to prion protein. **a**, Jpred4 prediction for the prion target region, outlined by a dashed box, shows a β-strand prediction for five residues. **b**,**g**, Circular dichroism data show that the PRI28 binder (**g**) and IL-2RG-30 (**g**) has helical secondary structure and is stable up to 95 °C. **c**, The design model of PRI22, designed using target sequence information alone. **d**, The BLI data revealed that the binding affinity of PRI22 is 1.88 μM (left), which improved to 80 nM after two-sided partial diffusion (right). **e**, The specificity test for prion binder PRI28 (Fig. 2i) against various amyloid target sequences showed that PRI28 is highly specific, with some cross-reactivity observed only with TEME106B. **f**, Jpred4 secondary structure prediction for the IL-2RG target region, with the designed sequence highlighted by a black dashed box. Within this region, a short β-strand (green) is predicted for five residues, indicating localized strand propensity.

We next sought to make binders of the prion protein (253 residues), which is primarily found in neuronal cells in mammals. Aggregated forms of this protein are linked to prion diseases, a group of transmissible neurodegenerative disorders^34,35^. The pathological hallmark of prion diseases is the conformational conversion of the native, monomeric cellular prion protein (PrP^C^) into a misfolded and aggregated form (PrP^Sc^) characterized by a cross-β structure^36–39^. To target the amyloid core region of the prion protein, we targeted an 8-residue sequence, VNITIKQH (positions 180–187), in a β-strand conformation (Extended Data Fig. 6a). Of 48 designs tested, the tightest, PRI28, had a binding affinity of 14 nM (Fig. 2i), with stability up to 95 °C and was highly specific (Extended Data Fig. 6b,e). Specifying the secondary structure of the target region as a β-strand resulted in binders with higher affinity than using the target sequence information alone (14 nM from secondary structure specification (PRI28) versus 1.88 μM from sequence input (PRI22), improving to 80 nM following partial diffusion; Fig. 2i and Extended Data Fig. 6c,d).

Signal transduction via cell-surface receptors is mediated by their intracellular domains, which contain long disordered regions^40,41^. Developing binders targeted at these domains would be broadly useful for colocalization imaging applications and for the modulation of receptor activation. IL-2RG (also known as CD132; 369 residues) is shared among the interleukin receptors for IL-2, IL-4, IL-7, IL-9, IL-15 and IL-21 (ref. ^42^). We targeted an amino acid sequence in IL-2RG (ERLCLVSEIP, positions 327–336) predicted to be disordered (Fig. 1A and Extended Data Fig. 6f) as a strand; of 94 selected designs, one design had a binding affinity of 493 nM. Through two-sided partial diffusion, we increased the binding affinity to 97 nM (IL-2RG-30; Fig. 2j), with thermostability up to 95 °C (Extended Data Fig. 6g).

## Structural analysis of designed complexes

**Fig. 3:**
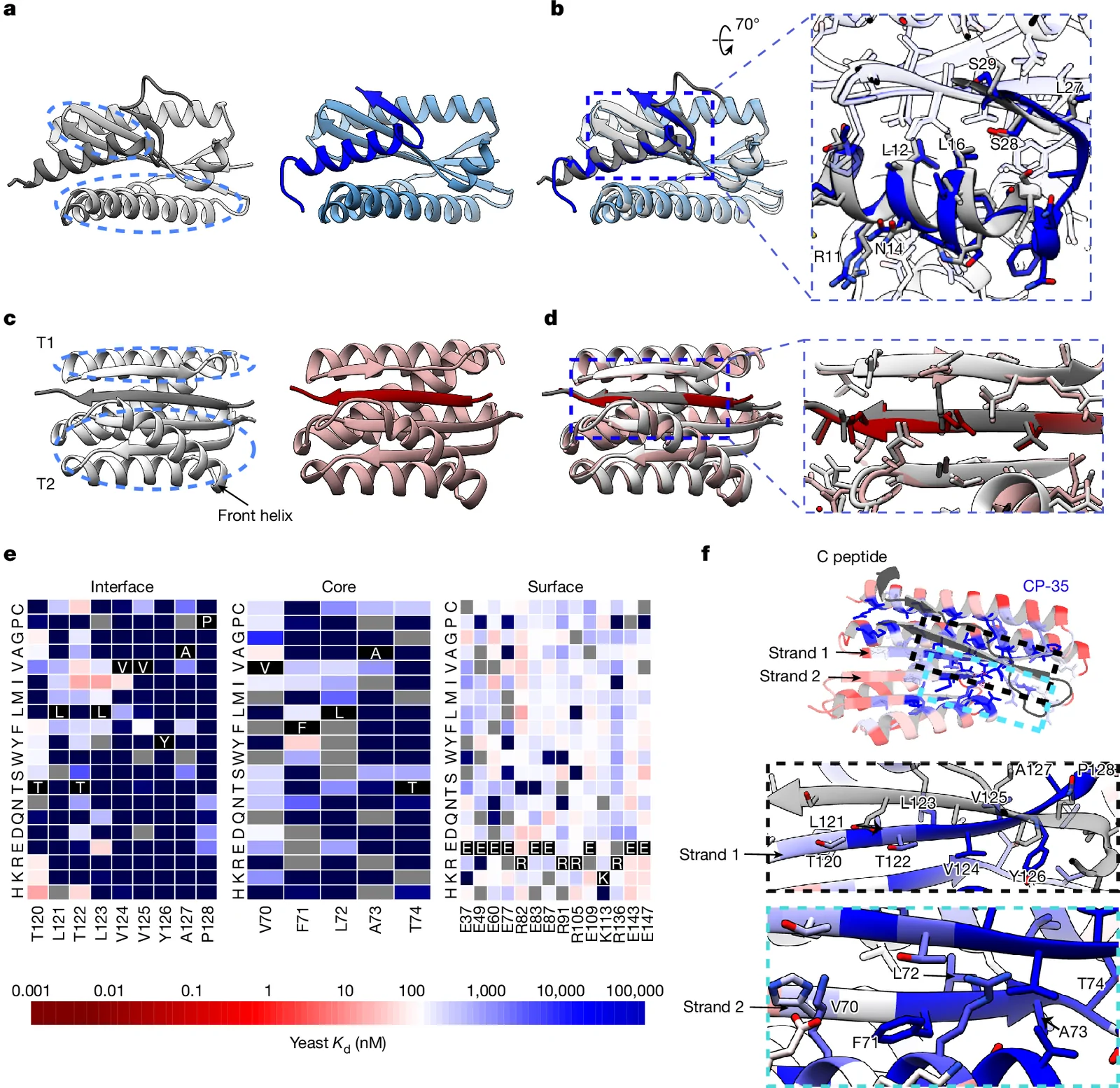
Structural characterization. **a**, Designed model of amylin-22_αβL_ in complex with amylin; amylin and designed binder are shown in dim grey and grey, respectively (left). Helical and strand segments forming the binding groove are highlighted by blue dashed ellipsoids. Right, crystal structure of the designed complex at 1.8 Å resolution, with the target and binder in blue and cornflower blue, respectively. **b**, Left, overlay of the design model and crystal structure of amylin-22_αβL_. Right, zoom-in on the designed interface. Binder proteins are rendered with 90% transparency, and key amylin residues are labelled. **c**, Left, design model of G3BP1-11. Two α/β-topologies (T1 and T2) of the binders are labelled. The front helix of T2 is denoted by a black arrow. Right, the crystal structure of G3BP1-11 at 2.4 Å resolution, with the target and binder proteins rendered in dark red and rosy brown, respectively. **d**, Left, overlay of the design model and the crystal structure of G3BP1-11. Right, zoom-in on the designed interface. **e**, Heatmaps representing C-peptide-binding *K*_d_ (nM) values for single mutations in the designed interface (left), core (middle) and surface (right). Depleted substitutions are in blue, beneficial substitutions are in red, and grey indicates the lost yeast strains. Strand 1 (interface), the right segment of strand 2 (core) and the exposed surface residues without contacts (surface) are highlighted (Extended Data Fig. 7e). **f**, Designed binders coloured by positional Shannon entropy from site saturation mutagenesis (top; conserved in blue and variable in red), and zoomed-in views of the design interface and core with C-peptide (bottom).

**Extended Data Fig. 7:**
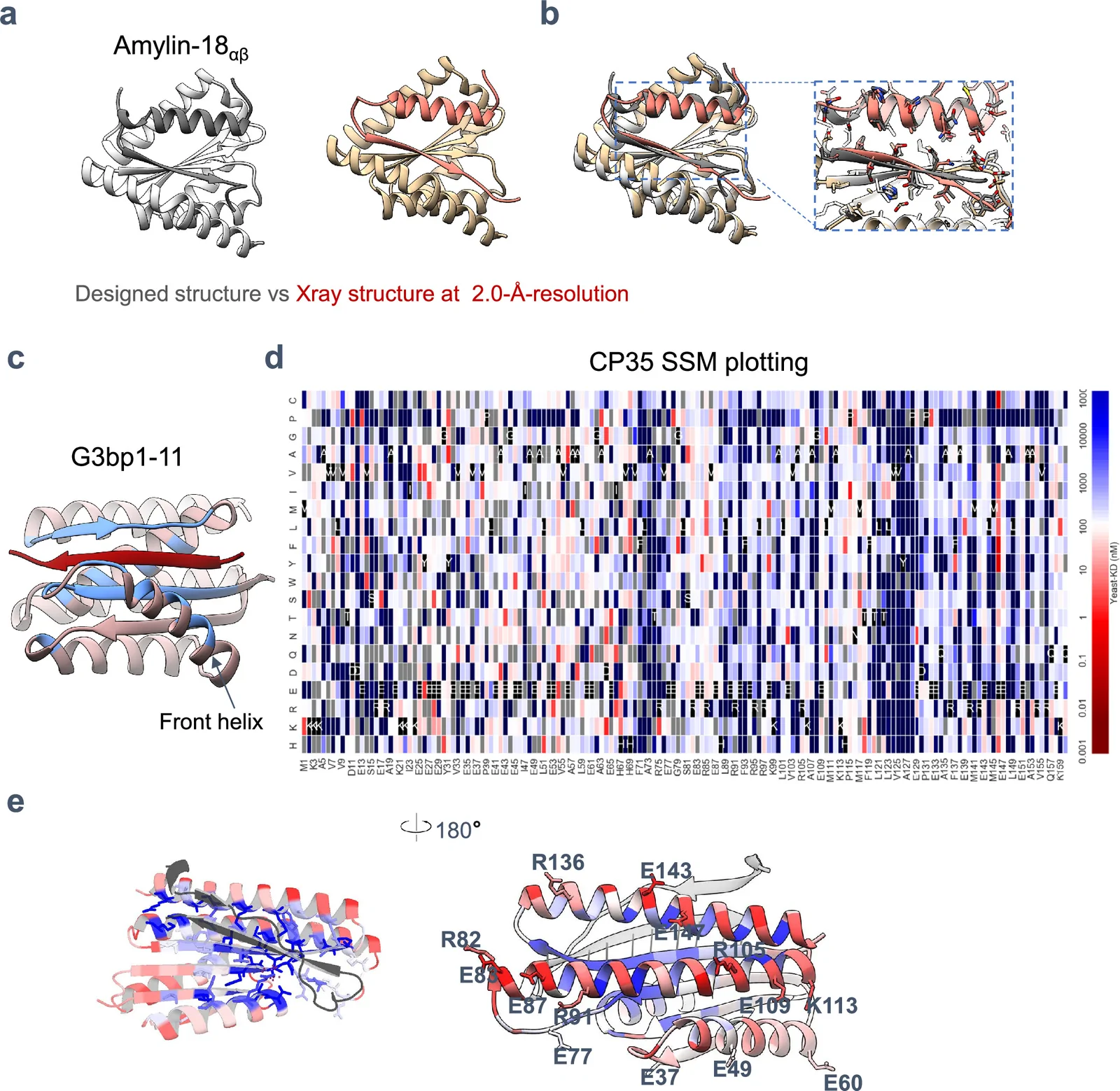
Crystal structure of Amylin-18_αβ_ and SSM analysis of CP-35. **a**, Left, the designed model of Amylin-18_αβ_, with target and binder proteins rendered in dim grey and grey, respectively. Right, the crystal structure of Amylin-18_αβ_ at 2.0 Å-resolution, with target and binder proteins rendered in salmon and tan, respectively. **b**, Left, the overlay of the design model and the crystal structure of Amylin-18_αβ_. Right, magnified views of the regions indicated with blue dotted frames in the left panel. The crystal structure of Amylin-18_αβ_ closely recapitulates the design model, with a Ca RMSD 0.741 Å for entire complex between design and crystal structure. The interface residues are nearly perfectly aligned with the design model structure, showing interface Ca and sidechain RMSD of 0.958 and 1.279, respectively. **c**, The crystal structure of G3bp1-11, positioned 4 Å away from the target on the binder, is marked in blue. **d**, Full SSM maps for the design of CP-35. **e**, Zoomed-in views of the residues presented in the surface region, as shown in Fig. 3e.

We obtained crystal structures of amylin-22_αβL_, amylin-18_αβ_ and G3BP1-11 in complexes with their target at 1.8, 2.0 and 2.4 Å resolution, respectively. For amylin-22_αβL_, the designed conformation comprises a helix, a strand and an unstructured loop (Fig. 3a, left). The amylin helix is embedded within a groove formed by the helix and strand segments of the binder. Adjacent to this, the amylin strand pairs with a corresponding strand of the binder. The amylin loop is predicted to be disordered based on the low per-residue AF2 pLDDT^25,43^ (Fig. 3a, left, and Extended Data Fig. 1c). In the crystal structure, the main helix and strand are well resolved and closely match the computational model; the disordered loop is, as anticipated, not resolved (Fig. 3a,b). The Ca root-mean-square deviation (RMSD) between the design model and the crystal structure over the backbone of the binder alone and over the backbone of the full complex excluding the missing loop of amylin are 0.96 and 2.04, respectively. The backbone and sidechains at the designed binder–target interface are also in close agreement between crystal structure and design model (Fig. 3b; the interface RSMDs for Ca and sidechain are 1.33 and 1.87, respectively). Amylin-18_αβ_, derived from the same parent as amylin-36_αβ_ via partial diffusion, closely matches the design model (Extended Data Fig. 7a,b).

In the G3BP1-11 design model, the target region is in a β-strand conformation and lies within a cleft formed by two α/β-structures, T1 and T2, in the designed binder, pairing with two adjacent strands (Fig. 3c). An additional helix in T2 also interacts with the target, potentially enhancing binding affinity and specificity (Fig. 3c and Extended Data Fig. 7c). The crystal structure of G3BP1-11 closely recapitulates the design model, with the targeted region clamped in a β-strand conformation (Fig. 3c,d; Ca RMSD of 0.8 Å for the entire complex between design and crystal structure) with the interface residues nearly perfectly aligned with the design model structure (Fig. 3c,d; interface Ca and sidechain RMSD are 0.86 and 2.29, respectively).

We were unable to solve crystal structures of the CP binders, so we instead obtained a lower-resolution structural footprint of the binding site by generating a site saturation mutagenesis library for CP-35 in which every residue was substituted with each of the 20 amino acids one at a time. Next-generation sequencing before and after FACS sorting for CP binding revealed that residues at the binding interface and protein core were largely conserved (Fig. 3e,f and Extended Data Fig. 7d,e), supporting the design model.

## Specificity of designed binders

**Extended Data Fig. 8:**
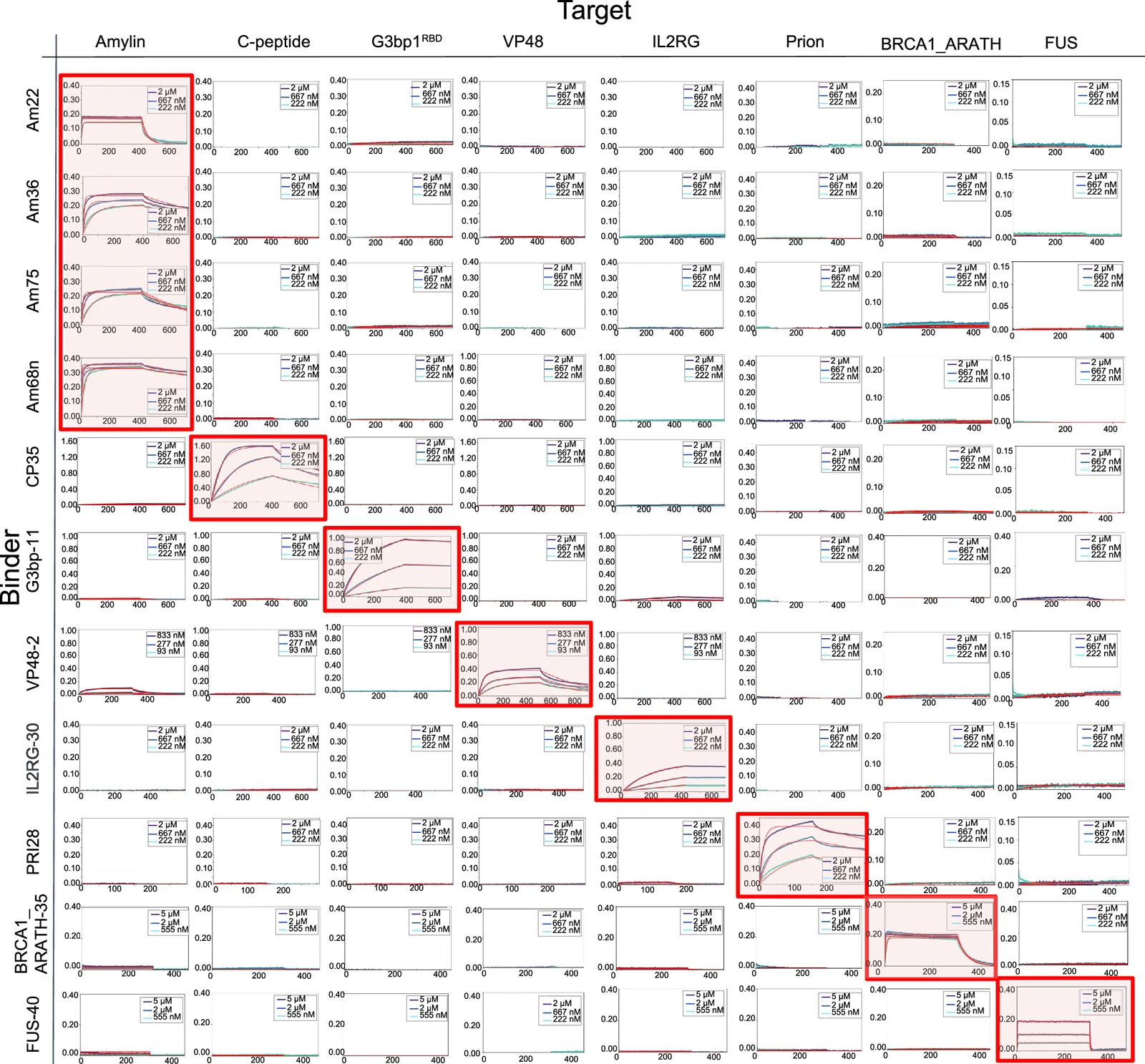
Designed binders show high specificity for their targets. Biotinylated peptides were immobilized onto octet streptavidin biosensors at equal densities and incubated with all binders in separate experiments at three concentrations. Amylin-68n_αβ_, −36_αβ_, −75_αα_, −22_αβL_ are abbreviated as Am68n, Am36, Am75 and Am22, respectively. The designed on-target interactions are indicated with a light red background.

We investigated the specificity of the binders by carrying out all-by-all binding experiments (Extended Data Fig. 8). BLI binding characterization of 11 binders against 8 targets showed that the designs had high specificity for their intended targets. Very weak off target binding was observed at high concentrations in two cases: binder VP48-2 weakly bound amylin above 800 nM, perhaps reflecting the approximately 50% helical content of both targets (specificity could potentially be further improved through another round of partial diffusion, or decreasing the helical percentage through secondary structure specification) and binder G3BP1-11 weakly bound IL-2RG at 2 µM.

## Binders colocalize with targets in cells

**Fig. 4:**
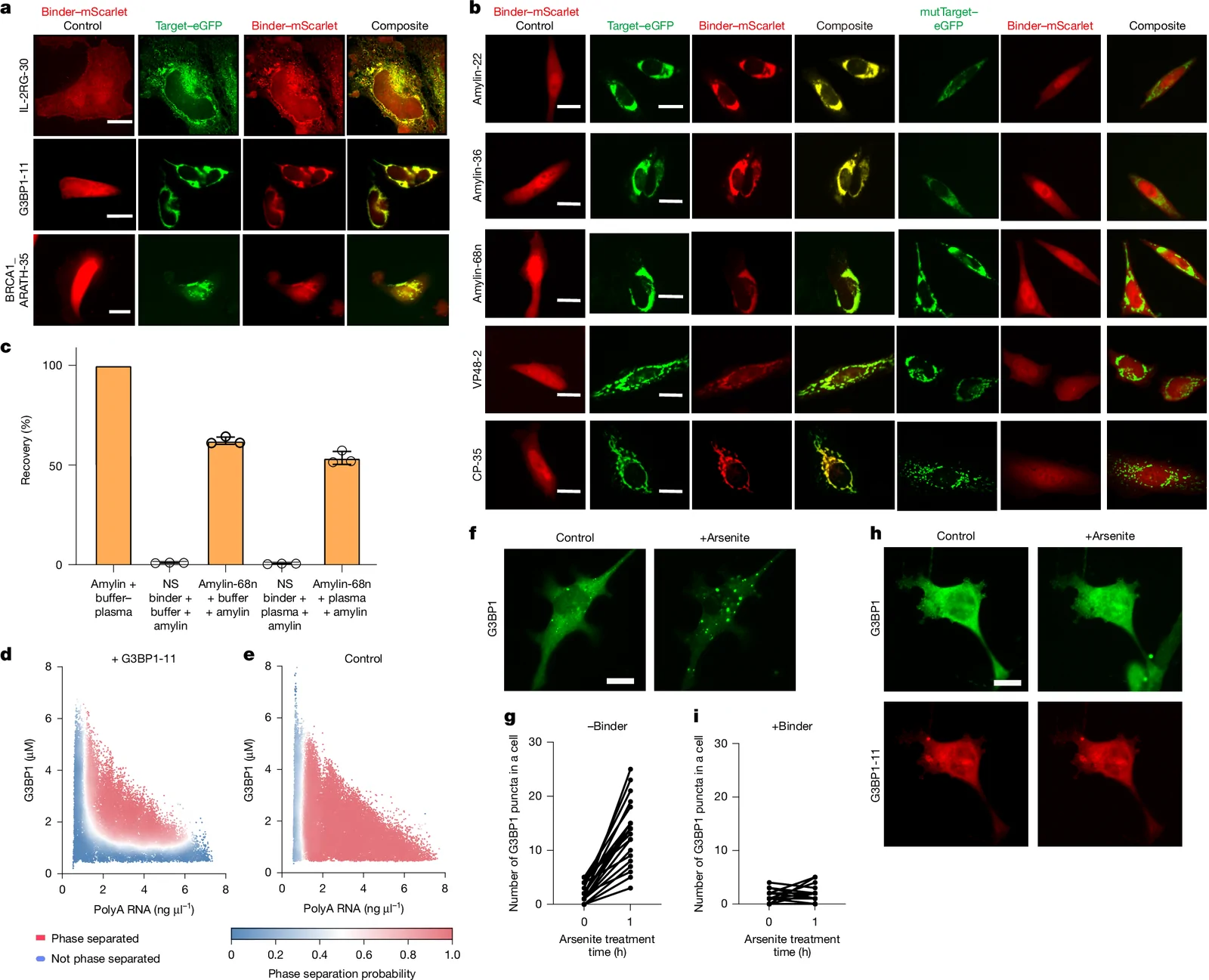
Designed binders bind to their targets in cells and binder G3BP1-11 modulates G3BP1 phase separation. **a**, Colocalization of IL-2RG-30, G3BP1-11 and BRCA1_ARATH-35 with their full-length targets in HeLa cells. Scale bars, 10 µm (top row) and 20 µm (middle and bottom rows). **b**, Colocalization of amylin-22, amylin-36, amylin-68n, VP48-2 and CP-35 with their targets. Scale bars, 20 µm. **c**, The LC–MS/MS recovery percent of amylin from phosphate-buffered saline (PBS)–0.1% 3-((3-cholamidopropyl) dimethylammonio)-1-propanesulfonate (CHAPS) buffer and EDTA-anticoagulated plasma was compared between BSA-blocked tosyl-activated bead, an off-target binder, and the binder amylin-68n. Percent recovery was calculated relative to the peak area of pure amylin in elution solvent (100% recovery). The data indicate mean ± s.d. (*n* = 3 technical replicates). **d**,**e**, Phase diagrams showing effects of G3BP1-11 (**d**) and control (**e**) on G3BP1–RNA phase separation across 0–8 ng μl^−1^ RNA. Red indicates phase-separated droplets, and blue indicates mixed states. **f**,**g**, Without binder, confocal images indicate that G3BP1 forms cytoplasmic puncta after 1 h of arsenite treatment (**f**). Quantification of G3BP1 puncta per cell (each point represents one cell; *n* = 20 cells per condition), showing a notable increase upon arsenite treatment, is also displayed (**g**). **h**,**i**, With the G3BP1-11 binder, arsenite treatment failed to induce a similar increase in puncta. Confocal images (**h**) show colocalization of G3BP1 (green) and the co-expressed mScarlet-tagged binder (red). Quantification revealed no markedly increase in puncta after arsenite treatment (**i**). Scale bars, 10 μm (**f**,**h**).

**Extended Data Fig. 9:**
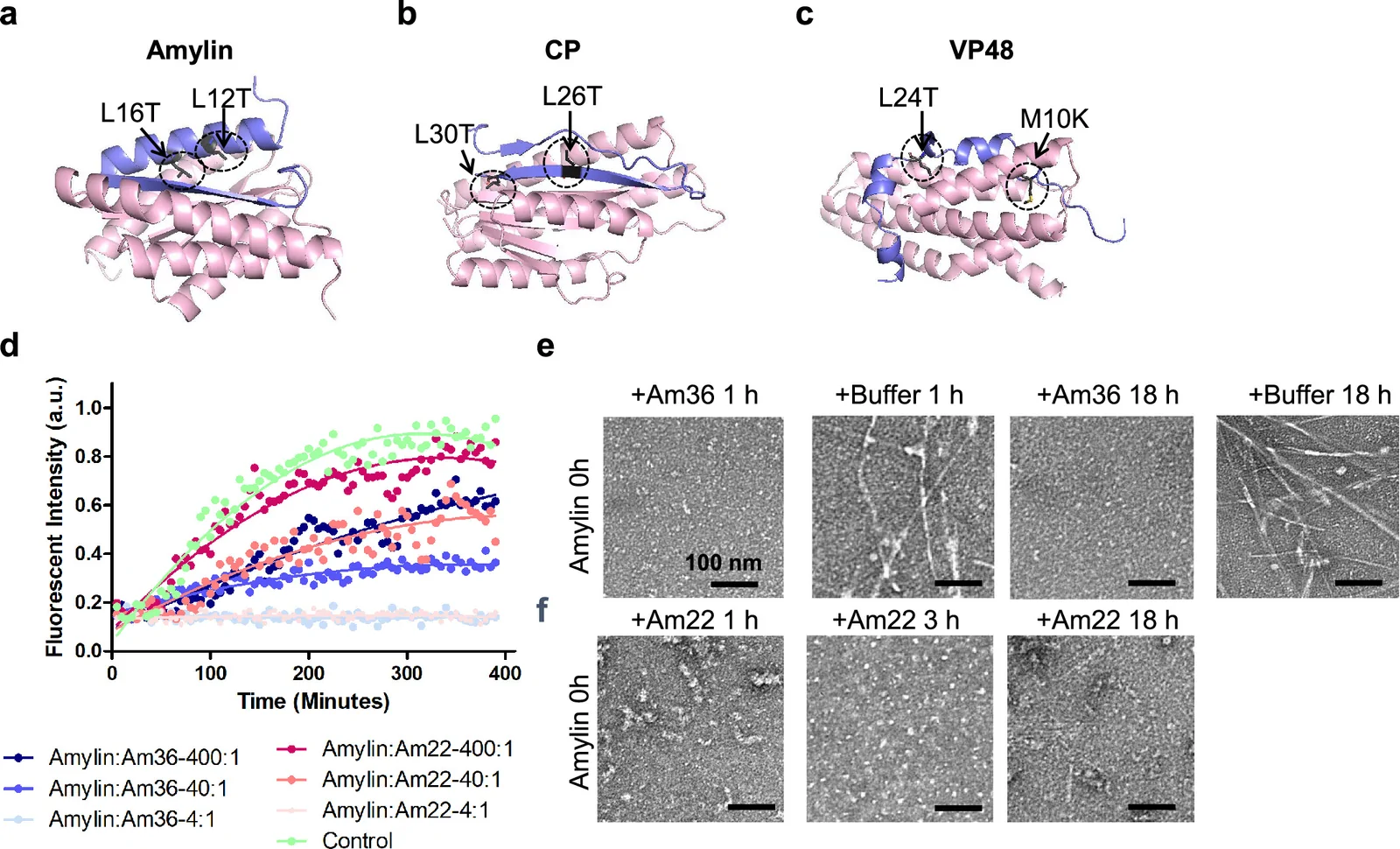
Point mutations at binder–target interfaces disrupt binding, while the designs inhibit Amylin fibril formation and dissociate existing fibrils. **a-c**, Designed complex structures of binders (light pink) in complex with their respective IDP targets (slate) amylin (**a**, binder Amy36 as representative), C-peptide (**b**), and VP48 (**c**). Point mutations introduced at the predicted interface are highlighted with arrows and dashed circles: L12T and L16T for amylin; L26T and L30T for C-peptide; and M10K and L24T for VP48. **d**, Amylin binders Amylin-22_αβL_ and Amylin-36_αβ_ inhibit fibril formation in a concentration-dependent manner. The initial concentration of Amylin monomer was 10 μM, with subsequent additions of binders at 2.5 μM, 0.25 μM, and 0.025 μM, establishing molar ratios of binder to Amylin of 1:4, 1:40, and 1:400, respectively. **e**,**f**, Negative stain electron microscopy images were taken of 40 μM Amylin monomer samples following the addition of 10 μM Amylin-36_αβ_ (**e**) and Amylin-22_αβL_ (**f**) at 1 h and 18 h, respectively. Scale bars, 100 nm.

To examine whether the designs could fold properly and bind to the full-length target proteins in mammalian cells, we performed fluorescence colocalization experiments in cultured cells for all designed binder–target pairs except for the prion binder, which was excluded due to safety concerns, and the FUS binders, whose binding affinities were not considered sufficient for cellular binding. The full-length target proteins were fused to eGFP and a mitochondrial localization signal, and the designed binders were fused to mScarlet; if the design binds to the target in cells, GFP and RFP will be colocalized in puncta corresponding to mitochondria. We observed clear colocalization of design with target for amylin-68n_αβ_, amylin-36_αβ_, amylin-22_αβL_, CP35, VP48-2, IL-2RG-30, G3BP1-11 and BRCA1_ARATH-35, indicating successful target engagement in cells (Fig. 4a,b; in the case of IL-2RG, we replaced the endogenous protein with a construct encoding IL-2RG fused to eGFP using CRISPR–Cas9). For short IDPs such as amylin, CP and VP48, we introduced two point mutations at the predicted interface on the target (Extended Data Fig. 9a–c), and found that these mutations disrupted colocalization, confirming interface-specific recognition. Collectively, our results indicate that the designed binders can bind to and colocalize with their targets in the cellular environment.

## Enrichment for LC–MS/MS detection

We explored the use of the amylin binder amylin-68n_αβ_ as a capture agent for immunoaffinity enrichment combined with liquid chromatography–tandem mass spectrometry (LC–MS/MS), a general platform for detecting low-abundance protein biomarkers in human serum^44^. Amylin recoveries using amylin-binder-conjugated beads from human plasma or the simplified PBS–CHAPS matrix^45^ using spiked amylin into serum (endogenous levels were too low for reliable detection) were 62.2% and 53.5%, respectively (Fig. 4c). Tighter binders generated through further optimization will probably be necessary to enrich endogenous amylin.

## G3BP1-11 disrupts stress granule assembly

**Extended Data Fig. 10:**
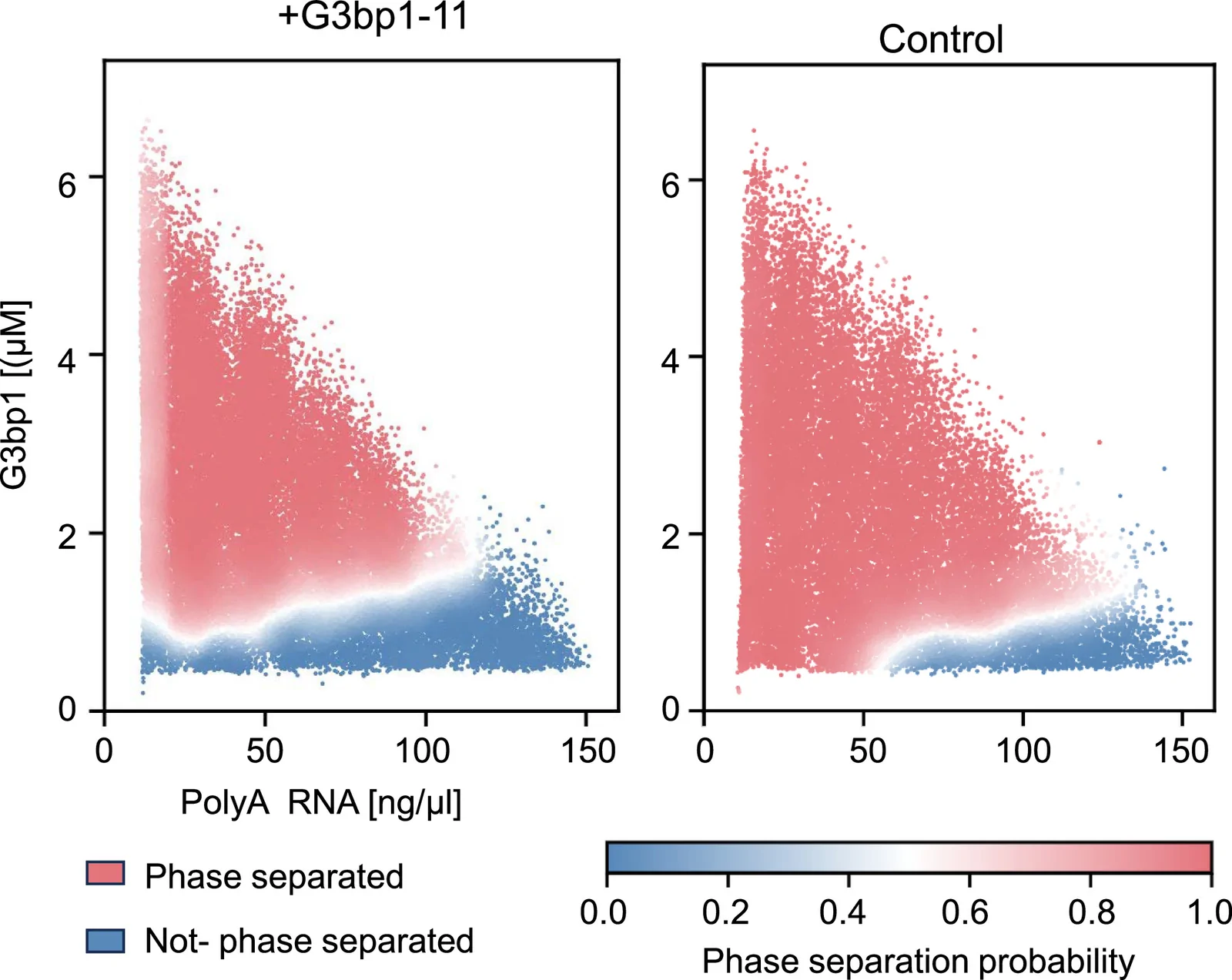
The effect of the binder on G3bp1 phase separation at higher polyA RNA concentrations. Phase diagrams comparing the effects of the G3BP1-11 binder (left) and control (right) on phase separation between G3BP1 and RNA over an RNA concentration range of 0–150 ng/μl.

To explore whether G3BP1-11 could disrupt or potentiate stress granule formation, we first tested the effects of the binders on phase separation in vitro. We used droplet microfluidics followed by imaging to assess G3BP1 phase separation at various concentrations of G3BP1 and polyA RNA. The phase diagrams with and without binder show that the G3BP1-11 design shifts the boundary of G3BP1 and polyA RNA phase separation; the effect of the binder was more pronounced at lower polyA RNA concentrations, requiring higher levels of G3BP1 for phase separation (Fig. 4d,e and Extended Data Fig. 10). In the presence of the binder, there was a visible phase boundary at 1 μM G3BP1 below which phase separation is not observed (Fig. 4d,e), probably resulting from G3BP1-11 binding to the C-terminal region of G3BP1 and blocking its interaction with RNA, which is important for G3BP1–RNA co-phase separation^33^.

We next investigated the function of the G3BP1 binder in cells by assessing G3BP1 puncta formation in response to arsenite-induced stress granules. In the absence of the binder (−binder), G3BP1 forms puncta upon arsenite treatment (Fig. 4f), with a notable increase in the number of puncta per cell (Fig. 4g), reflecting G3BP1 recruitment to stress granules under stress conditions. The G3BP1 binder interferes with this stress granule formation: arsenite treatment does not markedly increase G3BP1 puncta formation in the presence of the binder (Fig. 4h), with the number of G3BP1 puncta remaining low despite arsenite exposure (Fig. 4i).

## Designs inhibit and dissociate amylin fibrils

**Fig. 5:**
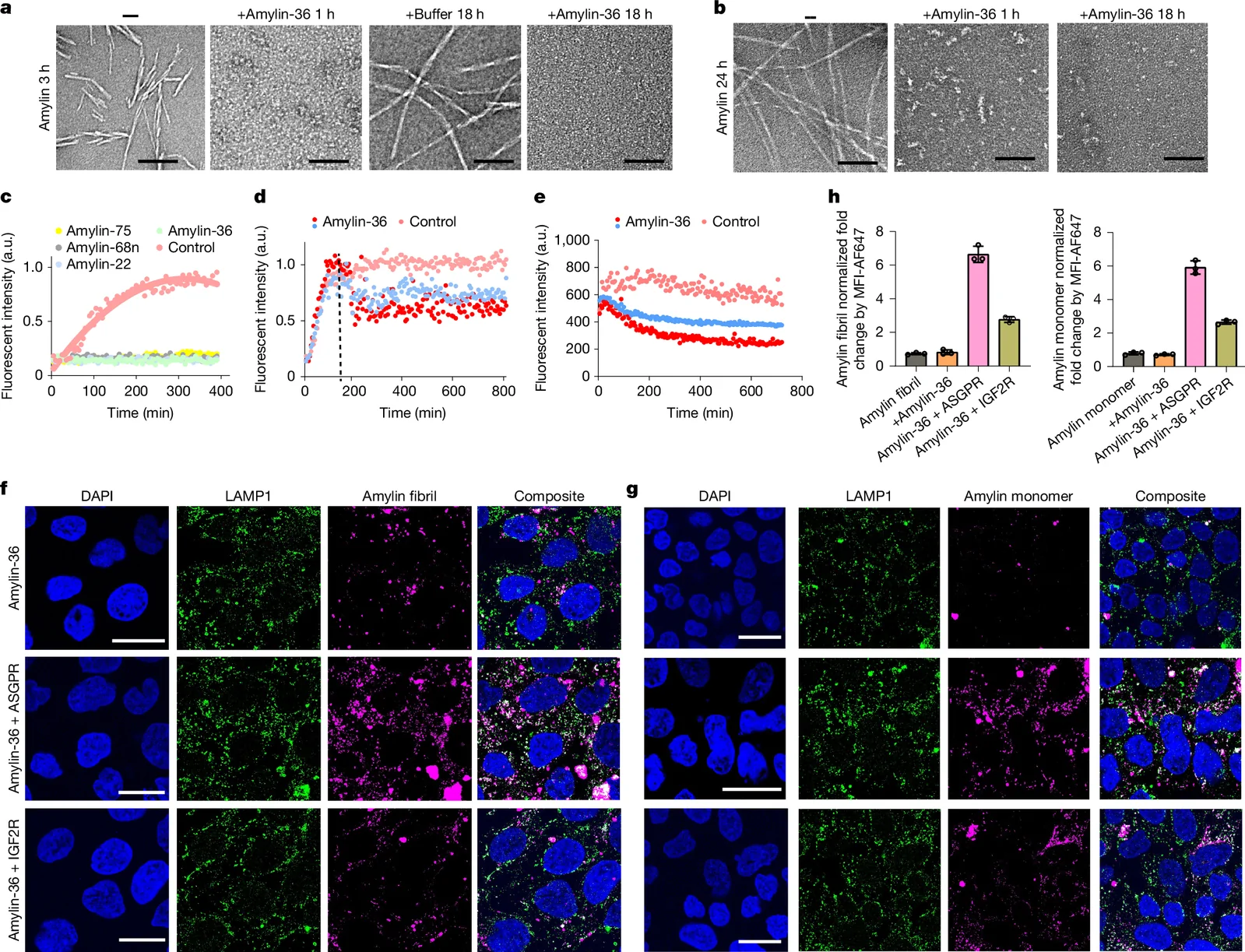
Amylin fibril disruption and degradation using designed binder. **a**,**b**, NS-EM visualization of fibril dissociation by amylin-36_αβ_ at both elongation (**a**) and mature (**b**) phases. Scale bars, 100 nm. **c**, The ThT assay revealed that all four binders could strongly inhibit fibril formation at a binder:amylin molar ratio of 1:4. **d**, Amylin-36_αβ_ could dissociate fibrils at the elongation phase in a concentration-dependent manner. Amylin-36_αβ_ was added at 3 h during the elongation phase of amylin fibrils (as marked by the dashed line), and the ThT assay was performed to monitor the process. The red and blue dots indicate amylin-36_αβ_:amylin molar ratios of 1:4 (10 μM binder) and 1:40 (1 μM binder), respectively. **e**, The ThT assay was performed after the mature amylin fibrils were formed for 24 h and, at the same time, amylin-36_αβ_ was added. The red and blue dots indicate that the amylin-36_αβ_:amylin molar ratios are 1:4 (10 μM binder) and 1:40 (1 μM binder), respectively. **f**,**g**, Confocal microscopy images of Hep3B cells treated with Alexa Fluor 647-labelled amylin fibrils (**f**; 24 h incubation at 37 °C) or monomers (**g**), in complex with amylin-36 (top), amylin-36–ASGPR (middle) or amylin-36–IGF2R (bottom). DAPI (blue) stains nuclei, LAMP1 (green) marks lysosomes, and amylin (magenta) is visualized via fluorophore-conjugated streptavidin. Composite panels (rightmost) show merged channels. Scale bars, 25 μm (**f**,**g**). **h**, Quantification of internalized amylin fibrils (left) and monomers (right) by flow cytometry; the median fluorescence intensity (MFI) was measured. Data are shown as mean ± s.d. from three biological replicates.

Amylin fibril formation is implicated in type 2 diabetes, in which the aggregation of amylin into insoluble fibrils contributes to islet amyloid deposition and β-cell dysfunction^46^. We investigated the effect of four binders on amylin fibril formation: amylin-68n_αβ_, amylin-36_αβ_, amylin-75_αα_ and amylin-22_αβL_. At a binder:amylin molar ratio of 1:4 (10 μM binder, 40 μM amylin), all binders completely inhibited fibril formation (Fig. 5c). Further tests with amylin-22_αβL_ and amylin-36_αβ_ at binder:amylin molar ratios of 1:4, 1:40 and 1:400 revealed a concentration-dependent retardation of fibril formation (Extended Data Fig. 9d). Inhibition of fibril formation was also observed by negative-stain electron microscopy (NS-EM). Addition of amylin-36_αβ_ blocked fibre formation at both 1 h and 18 h, whereas some short fibrils were observed 18 h post-addition of amylin-22_αβL_ (Extended Data Fig. 9e,f).

We next investigated whether the amylin binders were able to disaggregate pre-formed amylin fibrils. We generated short amylin fibrils by incubating amylin at 40 μM for 3 h at 37 °C to reach the elongation phase, and then incubated with 10 μM amylin-36_αβ_. NS-EM revealed no fibrillar structures after treatment with amylin-36_αβ_ at both 1 h and 18 h timepoints (Fig. 5a). Thioflavin T (ThT) assays with amylin-36_αβ_ added at the 3-h amylin fibre stage also showed fibre disassembly in a design concentration-dependent manner (Fig. 5d).

To test whether amylin-36_αβ_ could dissociate mature fibrils that had formed over 24 h at 10 μM, we incubated them with 10 μM of the binder. Small oligomers were still observed at 1 h, but were completely dissociated by 18 h (Fig. 5b). Fibril ThT fluorescence again decreased in a designed binder concentration-dependent manner (Fig. 5e).

## EndoTag fusion targets amylin to lysosomes

Amyloid plaques of amylin have been found in over 95% of patients with type II diabetes mellitus, mainly as extracellular deposits in the proximity of pancreatic β-cells^47^. When amylin fibrils lie between β-cells and capillary endothelial cells, they can impair the flow of nutrients (for example, glucose), leading to interference with the exocytosis of insulin from the secretory vesicles^47,48^. Endocytosis and lysosomal trafficking of cell-surface receptors can be triggered by designed endocytosis-triggering binding proteins (EndoTags) against multiple receptors including insulin-like growth factor 2 receptor (IGF2R) and asialoglycoprotein receptor (ASGPR)^49^. Fusion of these tags to soluble target protein binders leads to lysosomal trafficking and target degradation^49^.

We fused the amylin-specific binder amylin-36_αβ_ to ASGPR and IGF2R EndoTags to generate amylin-36–ASGPR and amylin-36–IGF2R. To evaluate internalization efficiency, we used commercially available amylin monomers with an N-terminal biotin modification. We pre-complexed 500 nM amylin-36–ASGPR or amylin-36–IGF2R with 500 nM fluorophore-labelled amylin monomers or fibrils. Hep3B cells were then treated with the resulting complexes for 20 h. Confocal imaging revealed substantial intracellular accumulation of amylin in a punctate pattern, suggesting vesicular localization; co-staining with lysosomal markers further confirmed localization to lysosomes. By contrast, treatment with amylin-36 alone (without EndoTag fusion) resulted in minimal intracellular signal (Fig. 5f,g). Flow cytometry analysis confirmed these findings, showing an approximately eightfold and fourfold increase in median fluorescence intensity associated with cells for amylin-36–ASGPR and amylin-36–IGF2R, respectively (Fig. 5h), compared with amylin-36 alone (the greater internalization mediated by ASGPR is consistent with previous LYTAC results^50^). These results demonstrate that both amylin monomers and fibrils can be effectively internalized and trafficked to lysosomes via EndoTags technology.

## Discussion

Our results demonstrate the power of RFdiffusion in designing binders for IDPs ranging from 31 to 941 amino acids in length in diverse conformations. The ability to target IDPs without specifying the target structure is important as such proteins have no single defined conformation. During the design process, the target protein samples a wide range of possible conformations as the designed binding protein diffuses around it; the co-folding of design and target effectively enables the selection of conformations particularly suitable for binding. The resulting binders are well-folded proteins that engage specific subregions of the target in defined conformations through an induced-fit mechanism. For shorter regions that can adopt β-strand-like conformations, the introduction of a secondary structure specification feature within the RFdiffusion model enables targeting of short regions in the β-strand conformation with higher specificity and binding affinity than with previous strand targeting designs generated using Rosetta^11^. Our RFdiffusion based method is complementary to the recently described logos method^51^ for targeting IDPs: because it is trained on the PDB which is rich in interactions between regular secondary structures, the former excels at designing binders to sequences that can be bound in partial helical and beta-strand conformations, whereas the latter is optimal for targeting IDRs with sequence patterns more favorable for binding in more extended conformations.

The binders and approaches described here should be broadly useful given the current difficulty in targeting IDPs and IDRs, and the important roles these have in both normal physiology and disease. For example, the amylin binders inhibit amylin fibre formation and dissociate pre-existing fibres, and mediate lysosomal trafficking of amylin monomers and fibrils. Likewise, the G3BP1 binder disrupts stress granule formation of G3BP1 in cells; targeting G3BP1 to regulate stress granule dynamics could serve as a tool for probing neurodegenerative diseases and other stress-related cellular processes. The designed binders bind to their targets in cells, as illustrated by the colocalization of IL-2RG-30, amylin-68n_αβ_, amylin-36_αβ_, amylin-22_αβL_, CP-35, VP48-2, G3BP1-11 and BRCA1_ARATH-35 with their respective targets. Binders to IL-2RG and other cytokine receptors open new avenues for modulating cytokine signalling in feedback loops for adoptive cell therapies and other applications. More broadly, the ability of our designed binders to engage targets in the cellular environment highlights their potential for modulating the localization, stability or function of IDPs in diverse biological contexts, with room for further optimization in applications requiring very high specificity.

## Methods

### Binder design using sequence input RFdiffusion

For each target, approximately 10,000–50,000 diffused designs were generated given only sequence input of the target using RFdiffusion (v1.1.0). The resulting library of backbones were sequence designed using ProteinMPNN (v1.0.1)^24^, followed by AF2 + initial guess^26^. Initial guess is the protocol in which the protein structure provided to the model as an initial guess is first converted to AlphaFold atom positions. These positions are then provided, along with the standard model inputs into the AlphaFold Model Runner. In the AlphaFold class of the AlphaFold code, on the first recycle, the prev_pos variable is initialized to the input AlphaFold atom positions as opposed to the standard initialization of all zeros^26^. The resulting designs were filtered based on interface pAE_interaction, pLDDT. The pAE of interaction (pAE_interaction) between the binder and the target was used to evaluate the confidence of the predicted interface. Lower pAE_interaction values indicate higher confidence in the relative positioning of the two proteins. The per-residue pLDDT score measures the confidence of AF2 in the local structure of the binder. In addition, AF2 monomer was performed using only the binder sequence to filter based on the monomer pLDDT of the binder and RMSD to the binder design model (Supplementary Table 2). Subsequently, FastRelax was executed to obtain Rosetta metrics^52^. The resulting binders were then further filtered based on criteria including contact_molecular_surface^53^, ddG^54^, SAP score^55^ and the numbers of hydrogen bonds. Specific filtering criteria were carefully selected to narrow down the set to 48–96 designs for each target.

### Two-sided partial diffusion to optimize binders

Partial diffusion enables the input structure to be noised only up to a user-specified timestep instead of completing the full noising schedule. The starting point of the denoising trajectory is therefore not a random distribution. Rather, it contains information about the input distribution resulting in denoised structures that are structurally similar to the input. Unlike one-sided directional partial diffusion, which solely diversifies the conformation of the binder while keeping the target fixed, two-sided partial diffusion allows simultaneous conformational changes in both the target and the binder. The input designs were subjected to 5–25 noising timesteps out of a total of 50 timesteps in the noising schedule, and subsequently denoised. Approximately 5–50,000 partially diffused designs were generated for each target. The resulting library of backbones were sequence designed using ProteinMPNN^24^, followed by AF2 + initial guess^26^. The resulting designs were filtered in the same way as the designs from the aforementioned sequence input diffusion process.

### Secondary structure specifications

To permit specification of the secondary structure (but not three-dimensional coordinates) of the target, a modified version of RFdiffusion was trained that permits specification of the secondary structure of a region, along with its sequence. The training strategy largely followed that used to train previous RFdiffusion models^5,12^, with some modifications. A summary is provided below.

For an overview of ‘base’ RFdiffusion training, Rfdiffusion^5^ is a denoising diffusion probabilistic model, which is fine-tuned from the RoseTTAFold structure prediction model^25,56^. In RFdiffusion, the N-Ca-C frame representation (translation and orientation) of protein backbones^25,57^ is used, and, over 200 discrete timesteps, these backbone frames are corrupted following a defined forwards noising process that noises these frames to distributions indistinguishable from random distributions (three-dimensional Gaussian distribution for translations and uniform SO(3) distribution for rotations). RFdiffusion is trained to reverse this noising process, predicting the true (X0) protein structure at each timestep of prediction (starting from randomly sampled translations and rotations). Successive predictions are used to ‘self-condition’ predictions through an inference trajectory, and mean squared error losses minimize the error between forwards and reverse processes. Full details of training are described in Watson et al.^5^.

For modifications to permit secondary structure specification of the target, as in the original RFdiffusion fine-tuned for protein binder design, RFdiffusion was trained 50% of the time on single chains from the PDB < 384 amino acids in length, and 50% on heterocomplexes. In the latter case, one chain (less than 250 amino acids in length) was designated the ‘binder’, and when necessary the other ‘target’ chain was radially cropped around the interface (to 384, which is the length of the binder residues). For single-chain examples, 20% of the time, the whole backbone was noised; in the other 80% of cases, 20–100% of the protein backbone was noised. For heterocomplex examples, the whole binder chain was noised. In addition, and in contrast to the original RFdiffusion model trained for protein binder design, up to 50% of the noised monomer structure had sequence provided in the noised region. For heterocomplexes, up to 50% of the target chain backbone was also noised, whereas its sequence was provided to RFdiffusion. This permits RFdiffusion to condition on the sequence of the target chain in the absence of three-dimensional structure.

To permit specification of the secondary structure of the target (when three-dimensional coordinates are not provided), secondary structure and ‘block adjacency’^5^ information were provided to RFdiffusion in exactly the manner described in Watson et al.^5^. In brief, 50% of the time, RFdiffusion was provided with a (partially masked; 0–75%) secondary structure of the example protein chain or heterocomplex, and (an independently sampled) 50% of the time a (partially masked; 0–75%) block adjacency of the protein chain or heterocomplex. In addition, 50% of the time, the whole interchain block adjacency was masked in heterocomplex examples. This permits RFdiffusion to condition on a (partially) pre-specified secondary structure (and/or adjacency information) of the target. This version of RFdiffusion was trained for seven epochs.

To design binders using RFdiffusion through secondary structure specification, for each target, approximately 10,000 diffused designs were generated through sequence input of the target with the additional secondary structure specification. The resulting library of backbones were sequence designed using ProteinMPNN^24^, followed by AF2 + initial guess^26^. The resulting designs were filtered in the same way as the designs from the aforementioned sequence input diffusion process.

### Backbone extension for VP48 binder design

During the design campaign, not all designs provided sufficient interactions to the whole sequence of the target, especially the loopy regions. To explore and guide RFdiffusion to make more interactions around certain regions, we selected 20 AF2 passing designed complexes from the round one design campaign, based on the above criteria and manual selection. For each base design, we requested RFdiffusion to extend the binder backbone with 10–20 amino acids from either N terminus, C terminus or both (depending on where the loopy region was located). This was done with the inpaint flavour published in the original RFdiffusion work^5^. Two thousand designs were performed each run, followed by the same MPNN and AF2 predictions as above.

### Computational filtering

Precise metrics cut-offs changed for each design campaign to get to an orderable set, but largely focused on interface pAE_interaction < 10, pLDDT > 90, number of hydrogen bonds > 11, RMSD < 0.5, sap score < 45 and Rosetta ddG < −40 (ref. ^26^).

### Computational time and hardware usage

A typical binder design task, generating an approximately 80–150 residue binder, each backbone design using RFdiffusion took approximately 25–30 s when run on a single NVIDIA RTX2080 or A4000 GPU, using one CPU core and approximately 8 GB of RAM. The subsequent sequence design step using ProteinMPNN was notably faster and less resource intensive, requiring less than 0.5 s per backbone on a standard CPU (for example, Intel Xeon E5-2680).

### Gene construction of designed binders

The designed protein sequences were optimized for expression in *Escherichia coli*. Linear DNA fragments (eBlocks, Integrated DNA Technologies) encoding design sequences included overhangs suitable for Golden Gate cloning into the LM670 vector (Addgene #191552) for protein expression in *E. coli*. LM670 is a modified expression vector containing a kanamycin resistance gene, a ccdB lethal gene between BsaI cut sites and a C-terminal hexahistidine, commonly referred to as His tag.

### Binding screening

For screening for all designs except the ones of partial diffusion design for amylin-68n (Fig. 2a), the designs were screened by BLI (method details described below). Linear gene fragments encoding binder design sequences were cloned into LM670 using Golden Gate assembly. Golden Gate subcloning reactions of binders were constructed in 96-well PCR plates in 4 µl volume. One microlitre reaction mixtures were then transformed into a chemically competent expression strain (BL21 (DE3)). After 1-h recovery in 100 µl SOC medium, the transformed cell suspensions were directly transferred into a 96-deep-well plate containing 900 µl of LB media with kanamycin. After overnight incubation in 37 °C, 100 μl of growth culture was inoculated into 96-deep-well plates containing 900 µl of auto-induction media (autoclaved TBII media supplemented with kanamycin, 2 mM MgSO_4_, 1 × 5,052). After overnight incubation (6 h at 37 °C followed by additional 18 h at 30 °C), cells were harvested by centrifugation (15 min at 4,000*g*). Bacteria were lysed for 15 min in 200 μl lysis buffer (1× BugBuster (70921-4, Millipore), 0.01 mg ml^−1^ DNAse, and 1 tablet of Pierce protease inhibitor tablet per 50 ml culture). Lysates were clarified by centrifugation at 4,000*g* for 10 min, before purification on Ni-charged MagBeads (L00295, Genscript; wash buffer (25 mM Tris pH 8.0, 300 mM NaCl and 30 mM imidazole) and elution buffer (25 mM Tris pH 8.0, 300 mM NaCl and 400 mM imidazole)). Subsequently, the elutions were directly subjected to a BLI test and the final concentration was approximately 1 μM. The designs exhibiting binding signals were subsequently analysed by BLI through titration.

For amylin-68n, the designs from partial diffusion were expressed and purified using the same way as mentioned above. In addition to the designs, plasmids expressing target peptide fused with sfGFP (no His tag) were transformed into BL21 (DE3) cells, and overnight outgrowths were cultured in 5 ml of LB media with kanamycin. After overnight incubation in 37 °C and 250 rpm, growth cultures were inoculated into 50 ml auto-induction media. After overnight incubation in 37 °C and 250 rpm, cells were harvested by centrifugation (15 min at 4,000*g*), then resuspended in 20 ml lysis buffer (25 mM Tris-HCl, 150 mM NaCl, 0.1 mg ml^−1^ lysozyme, 10 μg ml^−1^ DNAse I and 1 mM PMSF). Of lysate of each binder, 100 µl was mixed with 100 µl of lysate of target peptide fused with sfGFP and incubated at room temperature for 15 min for co-lysis and target binding to the binders. Mixed lysates were applied directly to a 100 µl bed of Ni-NTA agarose resin in a 96-well fritted plate equilibrated with a Tris wash buffer. After sample application and flow through, the resin was thoroughly washed, and samples were eluted in 200 µl of a Tris elution buffer containing 300 mM imidazole. All eluates were sterile filtered with a 96-well 0.22-µm filter plate (203940-100, Agilent) before size-exclusion chromatography. Protein binders were then analysed for target binding via sfGFP co-elution with the His-tagged binder. High-performance liquid chromatography (HPLC) analyses were conducted using an Agilent HPLC system (Agilent 1260 Infinity II Liquid Chromatography system). Co-lysates were run on a Superdex200 Increase 5/150 GL column (28990945, Cytiva) with buffer of 25 mM Tris-HCl and 150 mM NaCl. To assess the binding interaction between the target and the binder, we monitored the elution profile of sfGFP using an absorbance wavelength of 395 nm, alongside a simultaneous measurement at 280 nm for total protein content to determine the extent of overlap between 395 nm and 280 nm, which indicates the binding interaction.

### Medium-scale protein expression and purification

For further validation, the initial hits were expressed at the 50-ml scale via auto-induction for approximately 24 h, in which the first 6-h cultures were grown at 37 °C and the remaining time at 22 °C. Cultures were harvested at 4,000*g* for 10 min and resuspended in approximately 20 ml lysis buffer (25 mM Tris-HCl, 150 mM NaCl, 0.1 mg ml^−1^ lysozyme, 0.01 mg ml^−1^ DNAse, 1 mM PMSF and 1 tablet of Pierce protease inhibitor tablet per 50 ml culture). Sonication was performed with a four-prong head for 5 min total, 10-s pulse on–off at 80% amplitude. The resulting lysate was clarified by centrifugation at 14,000*g* for 30 min. Lysate supernatants were applied directly to a 1-ml bed of Ni-NTA agarose resin equilibrated. After sample application and flow through, the resin was thoroughly washed, and samples were eluted by an elution buffer containing 400 mM imidazole. After elution, protein samples were filtered and injected into an autosampler-equipped Akta pure system on a Superdex S75 Increase 10/300 GL column at room temperature. The size-exclusion chromatography running buffer was 25 mM Tris-HCl and 150 mM NaCl pH 8. Protein concentrations were determined by absorbance at 280 nm using a NanoDrop spectrophotometer (Thermo Scientific) using their extinction coefficients and molecular weights obtained from their amino acid sequences.

### BLI binding experiments

BLI experiments were performed on an Octet Red96 (ForteBio) instrument, with streptavidin-coated tips (18-5019, Sartorius). Buffer comprised 1X HBS-EP+ buffer (BR100669, Cytiva) supplemented with 0.1% w/v bovine serum albumin. Before target loading, each design was tested for binding against unloaded tips. Of biotinylated target protein, 50 nM was loaded on the tips for 50 s followed by a 60-s baseline measurement. After loading, all designs underwent a 60-s baseline, 300–1,200-s association and 200–800-s dissociation. Baseline measurements of unloaded tips were subtracted from their matched measurement of the loaded tip. The hits were taken forwards for further titration experiments, for which concentration, association and dissociation times were chosen based on apparent affinity from the single-point screen. Global kinetic fitting was used to determine *K*_d_ across the dilution series.

In the specificity test of the designed binders, final concentrations were 2, 0.667 and 0.222 μM for most binders; 0.833, 0.277 and 0.093 μM for VP48; and 5, 2 and 0.555 μM for BRCA1_ARATH-35 and FUS-40.

### Circular dichroism experiments

For circular dichroism experiments, designs were diluted to 0.4 mg ml^−1^ in 25 mM Tris-HCl and 150 mM NaCl. Spectra were acquired on a JASCO J-1500 circular dichroism spectrophotometer. Thermal melt analyses were performed between 25 °C and 95 °C, measuring circular dichroism at 222 nm. All reported measurements were acquired within the linear range of the instrument.

### Affinity enrichment of amylin analysed by LC–MS/MS

#### Bead preparation

Anti-amylin binder-coated beads were prepared by conjugating each amylin-targeted binder (amylin-68n) to paramagnetic M280 tosylactivated beads (Invitrogen). Each sample reaction conjugated 1 µg of binder to 225 µg of beads. Beads were blocked with a solution of 0.01% bovine serum albumin (BSA) in 0.2 M Tris to minimize nonspecific interactions. An off-target binder-conjugated bead was included for quantification of nonspecific binding. A BSA-blocked bead without a bound binder was used as a negative control, and an anti-GPVGPSGPPGK (GPVG) peptide monoclonal antibody-conjugated bead was used as a positive control for the affinity binding step.

#### Sample preparation

Human amylin peptide (non-amidated) was purchased from Anaspec and reconstituted to 2 mg ml^−1^ in dimethylsulfoxide (DMSO). A secondary peptide stock (diluted into 50 µM in 5% acetonitrile, 0.1% formic acid and 0.01% BSA in water) was reduced with dithiothreitol (10 mM final concentration) and alkylated with iodoacetamide (30 mM final concentration). Excess iodoacetamide was quenched with additional dithiothreitol (5 mM final added concentration). This solution was diluted to a working stock of 10 μM with dilution solvent. Aliquots of the working stock were made in 1.5-ml LoBind tubes and stored at −20 °C to avoid repeated freeze–thaw cycles.

#### Human specimens

Human plasma samples were composed of pooled de-identified leftover clinical samples obtained from the clinical laboratories at the University of Washington Medical Center. The use of de-identified leftover clinical samples was reviewed by the University of Washington Human Subjects Division (STUDY00013706).

#### Affinity enrichment

Amylin capture experiments were performed using three types of coupled beads (amlin-68n, an off-target binder and BSA blocked) in PBS containing 0.1% CHAPS as well as pooled normal human EDTA-anticoagulated plasma.

Samples were prepared by spiking the working stock of alkylated amylin to a final concentration of 20 nM in 100 µl of either PBS–CHAPS or pooled plasma. Additional PBS–CHAPS was added to each sample, followed by coupled beads. GPVG peptide and anti-GPVG monoclonal antibody-conjugated beads were added to each sample as a positive control. The mixtures were shaken for 1 h at 900 rpm and room temperature (Thermomixer, Eppendorf). The supernatant was removed and the beads were washed twice with 200 μl of PBS–CHAPS. Bound peptides were eluted in 50 µl of elution solvent (20% acetic acid, 10% acetonitrile, 10% DMSO and 0.001% BSA in water) with shaking for 8 min (900 rpm at room temperature). Each bead type (two anti-amylin binders, one off-target binder and one BSA blocked) was assessed in separate samples and each was prepared in triplicate.

Sample analysis was performed by LC–MS/MS using a Shimadzu Nexera LC-XR HPLC coupled to a Sciex 6500+ triple quadrupole tandem mass spectrometer in multiple reaction monitoring mode. Specifications for the liquid chromatography, mass spectrometer and multiple reaction monitoring methods are included in Supplementary Tables 3–5.

#### Data analysis

Data processing was performed with Skyline Daily (v23.1.1.459). Chromatographic peak area was calculated by summing the peak area of all transitions for each peptide. The chromatographic peak areas observed during blank (elution solvent) injections were subtracted as background from sample peak areas before performing further data reduction. The signal from BSA and GPVG beads was for quality control of the assay and evaluated before processing of the experimental data.

Seven types of samples were analysed:

1. Group A: alkylated amylin peptide spiked directly into elution solvent served as the reference peak area for 100% recovery of amylin peptide.
2. Group B: paramagnetic tosyl-activated beads conjugated to an off-target binder were incubated in PBS–CHAPS spiked with alkylated amylin. The peak area of this negative control was used to quantify nonspecific binding.
3. Group C: amylin-targeted binders conjugated to paramagnetic tosyl-activated beads were incubated in PBS–CHAPS spiked with alkylated amylin. The peak areas of these samples were used to quantify the percent recovery of amylin by affinity enrichment.
4. Group D: an off-target binder conjugated to paramagnetic tosyl-activated beads was incubated with unspiked plasma. The peak area of this negative control was used to quantify the nonspecific signal from beads binding to plasma components.
5. Group E: amylin-targeted binders conjugated to paramagnetic tosyl-activated beads were incubated with unspiked plasma. The peak areas observed in these samples were used to quantify the nonspecific signal from the binders binding to plasma components (that is, assuming no non-amidated amylin in normal plasma).
6. Group F: an off-target binder conjugated to paramagnetic tosyl-activated beads was incubated with spiked plasma. The peak area of this negative control was used to quantify nonspecific binding.
7. Group G: amylin-targeted binders conjugated to paramagnetic tosyl-activated beads were incubated with spiked plasma. The peak areas of these samples were used to quantify percent recovery of amylin by affinity enrichment.

The percent recovery of each binder-coated bead type was calculated using equation (1) provided in Supplementary Information. The percent recovery of each binder-coated bead type was analysed using Graph Pad Prism 8.

### Preparation of SSM libraries

Saturation mutagenesis (SSM) was performed on all designs to gain a better understanding of the peptide-binding modes. CP-35 was selected for detailed analysis due to its structural complexity and the high-quality SSM data obtained. For CP-35, we ordered a SSM library covering all the 159 amino acids. The chip-synthesized DNA oligos for the SSM library were then amplified and transformed to EBY100 yeast together with a linearized pETCON3 vector. Each SSM library was subjected to an expression sort first, in which the low-quality sequences due to chip-synthesizing defects or recombination errors were filtered out. The collected yeast population, which successfully expresses the designed mutants, were regrown and subjected to the next round of peptide-binding sorts. Two rounds of with-avidity sorts were applied at 1 μM concentration of CP followed by one round of without-avidity sorts with CP concentrations at 200 nM, 40 nM, 8 nM, 1.6 nM and 0.32 nM. The peptide-bound yeast populations were collected and sequenced using the Illumina NextSeq kit. The mutants were identified and compared with the mutants in the expression libraries. Enrichment analysis was used to identify beneficial mutants and provide information for interpreting the peptide-binding modes. For each mutant, the fraction of cells collected in each of the five titration sorts of decreasing concentration was measured. The sorting concentration 50, the concentration at which 50% of the expressing cells are collected, was calculated and plotted in heatmaps for SSM analysis.

### X-ray crystallography

We attempted to solve structures for all of our designs, but only the amylin and G3BP1 complexes successfully crystallized.

Crystallization experiments were conducted using the sitting drop vapour diffusion method.

Initial crystallization trials were set up in 200-nl drops using the 96-well plate format at 20 °C.

Crystallization plates were set up using a Mosquito LCP from SPT Labtech, then imaged using UVEX microscopes and UVEX PS-256 from JAN Scientific. Diffraction quality crystals formed in 0.1 M succinic acid, sodium phosphate monobasic monohydrate, glycine mixture at pH 6 and 30% w/v PEG 1000 for amylin-22. For G3BP1-11, diffraction quality crystals appeared in 0.05 M calcium chloride dihydrate, 0.1 M Bis-Tris pH 6.5, and 30% v/v polyethylene glycol monomethyl ether 550. For amylin-18_αβ_, diffraction quality crystals appeared in 3.2 M ammonium sulfate and 0.1 M citrate pH 5.0.

Diffraction data were collected at the National Synchrotron Light Source II on beamline 17-ID-1 (AMF) for amylin-18_αβ_ and amylin-22_αβL_. Diffraction data were collected at the Advanced Light Source beamline 821 for G3BP1-11. X-ray intensities and data reduction were evaluated and integrated using XDS^42^ and merged/scaled using Pointless/Aimless in the CCP4 program suite^43^. Structure determination and refinement starting phases were obtained by molecular replacement using Phaser (v2.5.0)^44^ using the designed model for the structures. Following molecular replacement, the models were improved using phenix.autobuild, with rebuild-in-place to false and using simulated annealing. Structures were refined in Phenix (v1.21.1_5286)^45^. Model building was performed using Coot (v0.9.8.7)^46^. The final model was evaluated using Molprobity (v4.5.2)^47^. Data collection and refinement statistics have been recorded in Extended Data Table 1. Data deposition, atomic coordinates and structure factors reported in this paper have been deposited in the PDB (http://www.rcsb.org/) with the accession codes 9CC5, 9CC6 and 9NZH, respectively.

We used PyMOL (v2.4.0) and UCSF Chimera (v1.14) for generating figures.

### Cell culture

HeLa cells (from the American Type Culture Collection) were cultured in DMEM (11965-092, Gibco) at 37 °C in a humidified atmosphere containing 5% CO_2_, supplemented with 10% (v/v) FetalClone II serum (SH3006603, Cytiva) and 1% penicillin–streptomycin (15140122, Thermo Fisher).

### CRISPR–Cas9 knockout of IL2RG

Pooled *IL2RG*-knockout HeLa cells were generated using the Gene Knockout kit V2 from Synthego, using multi-guide single guide RNA targeting *IL-2RG* (guide 1: CAUACCAAUAAUGCAGAGUG guide 2: UCGAGUACAUGAAUUGCACU and guide 3: GAAACACUGAGGGAGUCAGU). The ribonucleoprotein complex with a ratio of 4.5:1 of single guide RNA and Cas9 was delivered following the protocol of the SE Cell Line 4D-Nucleofector X Kit S (V4XC-1032, Lonza), using the nucleofection program CN-114 on the Lonza 4D X unit.

### Transient transfection

Plasmids for binder–mScarlet, IL-2RG–eGFP, and other target-eGFP-mito-tag were either synthesized and cloned by Genscript or constructed in-house. HeLa cells were seeded at 70–80% confluency in a chambered coverslip with 18 wells (81816, ibidi). At the same time, HeLa cells were reverse transfected using Lipofectamine 3000 transfection reagent (L3000008, Thermo Fisher) according to the manufacturer’s protocol.

### Fluorescence imaging

Four-colour, 3D images were acquired with a commercial OMX-SR system (GE Healthcare). Toptica diode lasers with excitation at 488 nm and 568 nm were used. Emission was collected on three separate PCO.edge sCMOS cameras using an Olympus ×60 1.42 NA plan apochromat oil immersion lens. Images (512 × 512; pixel size of 6.5 μm) were captured with no binning. Acquisition was controlled with AcquireSR Acquisition control software. *Z*-stacks were collected with a step size of 250 nm. Images were deconvolved in SoftWoRx 7.0.0 (GE Healthcare) using the ratio method and 200-nm noise filtering. Images from different colour channels were registered in SoftWoRx using parameters generated from a gold grid registration slide (GE Healthcare). 

For imaging stress granules, cells were washed twice with FluoroBrite DMEM imaging media and subsequently imaged in the same media in the dark at room temperature. Epifluorescence imaging was performed on a Yokogawa CSU-X1 spinning dish confocal microscope with either a Lumencor Celesta light engine with seven laser lines (408, 445, 473, 518, 545, 635 and 750 nm) or a Nikon LUN-F XL laser launch with four solid-state lasers (405, 488, 561 and 640 nm), ×40/0.95 NA objective or ×60/1.4 NA oil immersion objective and a Hamamatsu ORCA-Fusion scientific CMOS camera, both controlled by NIS Elements 5.30 software (Nikon). The following laser and filter combinations (centre/bandwidth) were used: excitation of 473 nm and emission of 525/36 nm for GFP, and excitation of 545 nm and emission of 605/52 nm for RFP. Exposure times were 500 ms for all channels, with no emission gain set and no neutral density filter added. All epifluorescence experiments were subsequently analysed using ImageJ (v1.54p). Brightfield images were acquired on the ZOE Fluorescent Cell Imager (Bio-Rad).

### ThT fluorescence assay

Amylin fibrils at various growth stages (0, 3 and 24 h) were adequately mixed with ThT at a molar ratio of 1:1 and added into 96-well-plates containing different types and concentrations of binders (amylin-75, amylin-36, amylin-22 and amylin-68n). The samples were then incubated at 37 °C for 1–18 h with 600 rpm orbital shaking. ThT fluorescence signals were measured using a Thermo Varioskan Flash Multi Detection Microplate Reader (0 and 3 h) or a Perkin Elmer EnSight Multifunctional Microplate Reader (24 h) with excitation wavelength at 440 nm and an emission wavelength at 482 nm.

### NS-EM experiment

Samples for NS-EM were dropped onto freshly glow-discharged carbon-coated copper grids and incubated for 1 min, and excess sample was removed by blotting on filter paper. The grids were then stained with 2% (w/v) uranyl acetate for 1 min, and excess uranyl acetate was blotted off. Finally, the grids were examined using a Tecnai Spirit transmission electron microscope (FEI) at an acceleration voltage of 120 kV.

### Lysosomal trafficking of amylin monomers and fibrils

HEP3B cells (obtained from the American Type Culture Collection) were plated onto eight-well Labtek slides overnight. On the day of the treatment, biotinylated amylin monomers or fibrils were complexed with Alexa Fluor 647-labelled streptavidin (Thermo Fisher) at 500 nM. These were then pre-complexed with amylin-36 or amylin-36-EndoTags, and cells were treated with this complex for 20 h. Cells were then fixed with 4% paraformaldehyde, permeabilized and stained with LAMP1 monoclonal antibody (H4A3), Alexa Fluor 488 (MA5-18121, Thermo Fisher; 1:200 dilution), followed by goat anti-mouse IgG (H + L) cross-adsorbed secondary antibody, Alexa Fluor 488 (A-11001, Thermo Fisher; 1:500 dilution) and counterstained with DAPI. Cells were washed with DPBS and imaged with a Nikon A1R confocal microscope using a Plan Fluor ×60, 1.30 NA oil objective. The following laser settings were used: 405-nm violet laser, 488-nm blue laser and 639-nm red laser. Quantification of internalized amylin fibrils (left) and monomers (right) by flow cytometry was analysed using Graph Pad Prism 8. Flow cytometry was performed in Attune NxT flow cytometer (Thermo Fisher). The data were analysed in FlowJo (v9) software. The FACS sequential gating/sorting strategy is shown in Supplementary Fig. 1.

### PhaseScan

Droplet microfluidic experiments using PhaseScan^58^ were perfomed as previously described^59,60^.

Experiments were conducted under physiological conditions with 150 mM KCl and 50 mM Tris-HCl at pH 7.4. In all cases, 2% w/v PEG 10 K (Thermo Fisher Scientific) was added to the solutions. A 10 µM solution of G3BP1-emerald in physiological buffer was prepared by diluting the protein from a stock solution in 1 M KCl and 50 mM Tris pH 7.4. In addition, a stock solution of the binder G3BP1-11 was prepared at 20 µM in physiological buffer. A 200 ng µl^−1^ polyA RNA (Merck) solution, labelled with 3 µM Alexa Fluor 647 for concentration measurement, was also prepared. For the microfluidic experiment, four aqueous solutions containing protein, binder, RNA and buffer, along with an oil solution for droplet generation (HFE-7500 mechanical oil with 1.2% Bio-RAN), were used. These solutions were loaded into five separate inlets on a microfluidic chip via pressure control pumps (LineUp Flow EZ, Fluigent). The three aqueous solutions were mixed in a single channel before reaching the droplet junction, where droplets were formed by oil flow at 100 µl h^−1^. By varying the flow rates of the protein, RNA and buffer solutions between 5 and 54 µl h^−1^ while maintaining a constant flow of 21 µl h^−1^ for the binder (0 µl h^−1^ for the control), droplets of uniform size and binder concentration but varying protein and RNA concentrations were generated. These droplets were incubated for 4 min in the microfluidic chip as they moved through the incubation channel before entering a wider imaging chamber, where their flow slowed, allowing imaging. Droplets were imaged in continuous flow every 4 s using an openFrame epifluorescent microscope (Cairn Research) equipped with a ×10 air objective (Nikon CFI Plan Fluor) and a dichroic filter set (Cairn Research) to simultaneously capture two wavelengths (488 nm and 647 nm). Crosstalk calibration images were acquired by flowing single-dye droplets through the chip, with fluorescence in the other two channels used for crosstalk correction. Microscopic images of both phase-separated and homogeneous droplets were analysed using a custom Python script (Python v3.9.7). Droplets were identified through circle detection and filtered based on shape and radius to exclude erroneous detections. For each wavelength, fluorescence intensity — after illumination background subtraction — was mapped to a linear intensity–concentration fit, defined from the 1st to 99th percentile of fluorescence intensity relative to stock concentration. A convolutional neural network, trained on human-annotated data, classified droplets as either phase separated or homogeneous. Phase diagrams were generated, with each data point representing an individual droplet. Colouring reflects the local average of phase-separation classification in droplets with similar compositions, providing a consensus measurement of phase-separation probability across phase space.

### Statistics and reproducibility

All experiments, including those shown in Figs. 5a,b and 6a,b, were independently repeated at least three times with similar results.

### Reporting summary

Further information on research design is available in the Nature Portfolio Reporting Summary linked to this article.

## Online content

Any methods, additional references, Nature Portfolio reporting summaries, source data, extended data, supplementary information, acknowledgements, peer review information; details of author contributions and competing interests; and statements of data and code availability are available at https://doi.org/10.1038/s41586-025-09248-9.

## Supplementary information

Supplementary Information Supplementary Figure 1 and Supplementary Tables 1-5.

Reporting Summary

Supplementary Video 1 Sequence input diffusion trajectory for the fully diffused Amylin-binder complex.

## Figures and Tables

**Extended Data Table 1:**
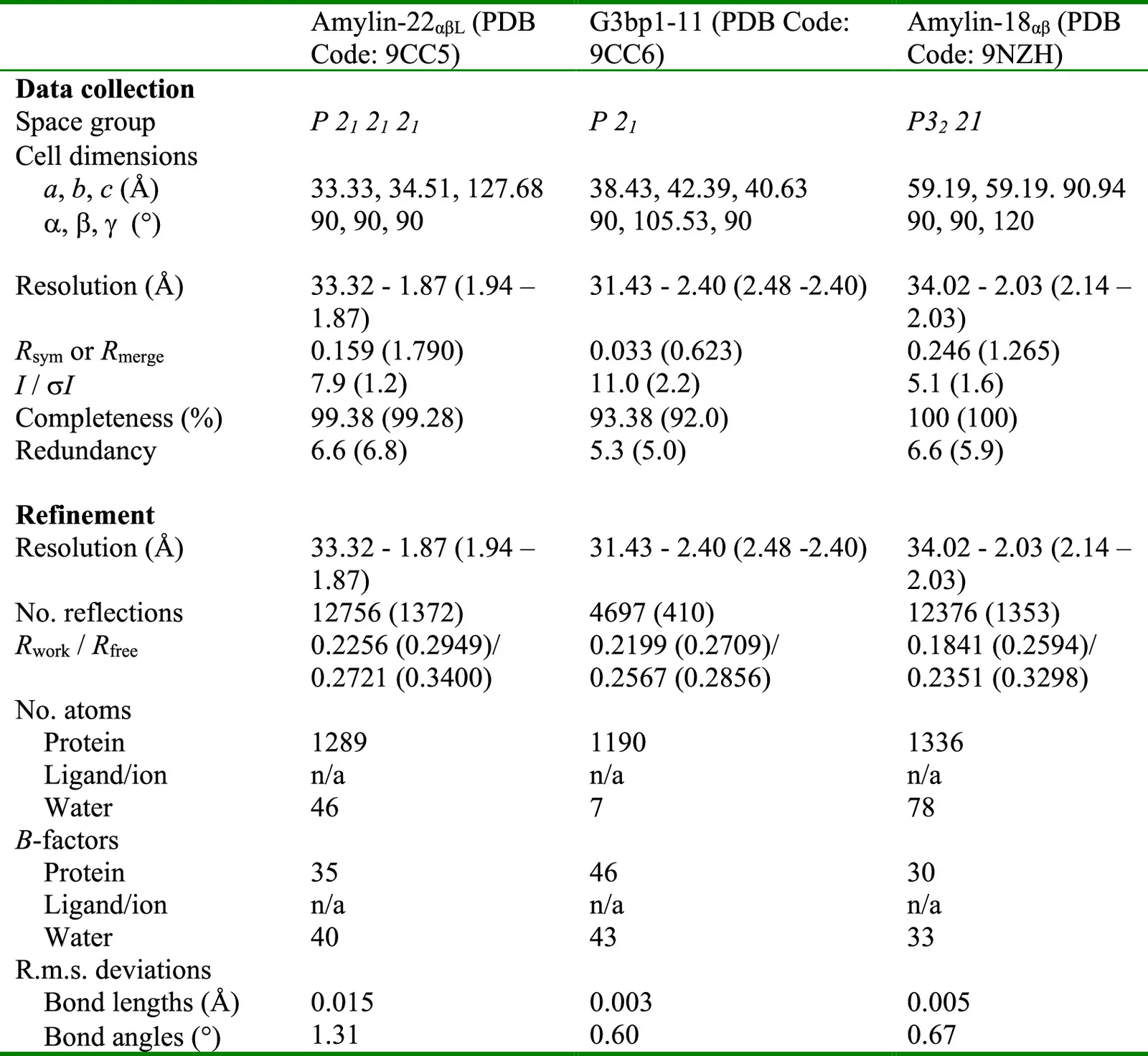
Crystallographic data collection and refinement

*Single crystal used for each data collection. *Values in parentheses are for highest-resolution shell.

## Data Availability

All data are available in the main text or as Supplementary Information. Crystal structures of amylin-18_αβ_, amylin-22_αβL_ and G3BP1-11 have been deposited in the PDB, with the accession IDs 9NZH, 9CC5 and 9CC6, respectively. Sequences of the binders described in this paper are in Supplementary Table 1. Design models are available at Zenodo: https://doi.org/10.5281/zenodo.16163643.

## Code availability

Code explanation and examples of binder design using RFdiffusion can be found on GitHub (https://github.com/RosettaCommons/RFdiffusion). The relevant code examples can be found in the ‘examples’ folder for reference: examples/design_ppi_flexible_peptide.sh for sequence input diffusion; examples/design_ppi_flexible_peptide_with_secondarystructure_specification.sh for secondary structure specification; and examples/design_partialdiffusion_withseq.sh for two-sided partial diffusion. The model weights used in this work (InpaintSeq_ckpt.pt and InpaintSeq_Fold_ckpt.pt) have been mirrored on Zenodo^61^ (https://doi.org/10.5281/zenodo.15453428) to ensure accessibility and reproducibility. The sequence input diffusion protocol uses both InpaintSeq_ckpt.pt and InpaintSeq_Fold_ckpt.pt; the latter biases the target conformation towards strand formation. Secondary structure specification uses InpaintSeq_Fold_ckpt.pt. Two-sided partial diffusion uses InpaintSeq_ckpt.pt. In addition, the code has also been deposited in Code Ocean^62^ (Capsule title: Diffusing protein binders to intrinsically disordered proteins; available at https://doi.org/10.24433/CO.0868770.v1).
