# Supplementary material for: Diffusing protein binders to intrinsically disordered proteins
Source: Nature. 2025 Jul 30;644(8077):809–17. doi: 10.1038/s41586-025-09248-9 (PMC12367549; doi:10.1038/s41586-025-09248-9)
Supplement: Supplementary file 1 [file 41586_2025_9248_MOESM1_ESM.pdf]

nature portfolio

https://doi.org/10.1038/s41586-025-09248-9

Supplementary information

Diffusing protein binders to intrinsically disordered proteins

In the format provided by the authors and unedited

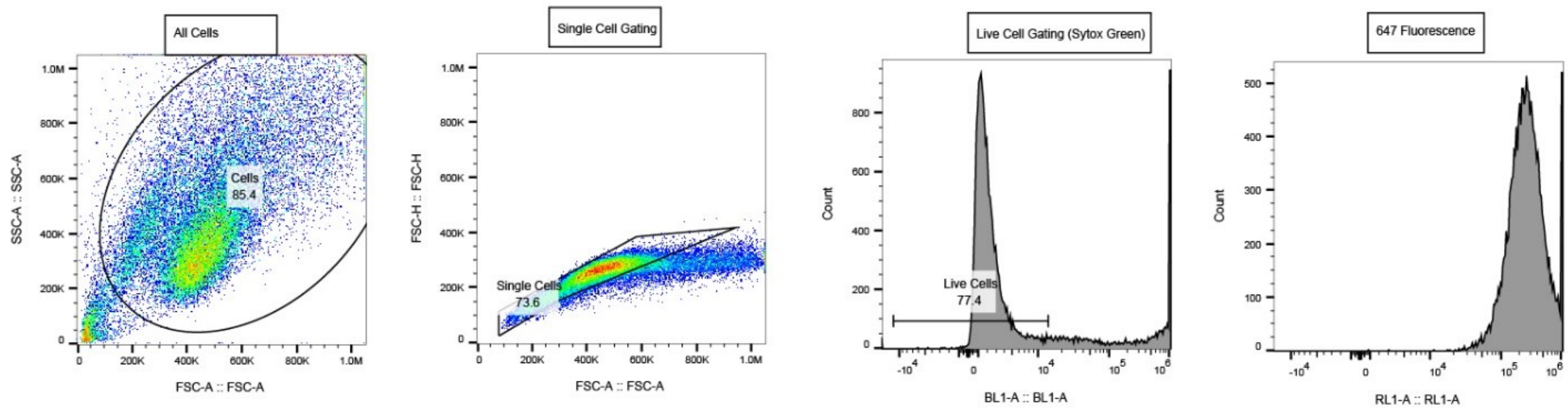

Supplementary Figure 1 Flow cytometry gating strategy related to Figure 6h: gating for all cells, single cells, live cells, and 647 fluorescence.

Supplementary Tables

IDP name	Targeted Sequence	Position	Binder sequence
Amylin	KCNTATCATQRLANFLVHSSNNFGAILSSTNVGSNTY (37)	Full length	amylin-68n$_{\alpha\beta}$: DMEEKIKKAKEEINKLLEKIVEWNKAGEKVELITVTGGIDTMPETLRRAAELIRKSGAKAVVVAGIEKVERSDPSAPSIEELLDEVADEIARLVRELGAEVVVVVDDSFSEEEIERLRERLAAEGAEVTVFSTSVVNEALE
			amylin-36$_{\alpha\beta}$: DMTEEEKKIIEDAKEEAEKLAPPFKKEVEESGKIPIHLINIFGWEEHKEAVKKASIESAKVMKDRAGVKGVIVLVSDPKDKEKAKEFAEEIAKELEEKLGVPVISLAHDSSEESIEKAKEEAKTQLEKLIAKIKGEETKP
			amylin-75$_{\alpha\alpha}$: EEEKEKLRKEAKEKTEKIIKDMEKQGYTLNEEQKEALENATFIVLAIDEANKRGEDVLSKLTTALKTASPEVQEQIATLLILVAEYLGLDVETIEKVVNEALEKAREEKK
			amylin-22$_{\alpha\beta L}$: SLIVAVASPVVVAAHSPEDEERAEKEAERLRRRFAEELRKKGFEVVELDEETDEELRRWLTKAIREATQAPTQEEFNQAVAEAIEKALERIEEIARRRHPDREVAAVLTVAVVHDGEVIATIFASPRLREALK
C peptide	EAEDLQVGQVELGGGPGAGSLQPLALEGSLQ (31)	Full length	CP35: MKKKAVVLVTDDEVSRERAEKKIKEYEEEGYEVEEVEVPLEAEGEEIAEALEEAVEAALEEGADEIHVHVFLATRVEEGLSREERVEHLEREFERLREKALEVARKAGELMAKHPNMTFTLTLVVYAPEDPEEQARFAERMRELMEEALREAARVRQEKQ
VP48	DALDDFDLDMLGSDALDDFDLDMLPADALDDFDLDMLGS (39)	Full length	VP48-2: VEDLQRWIDELLKLRERSRTRPVTREEFERAARLLALVYAVIVYTMAKLFLEAAKTGDKEKLEKAEKLLEIAEKIVKTIKSYTENPEARLHVLATIYEASEPYIEKALKLLEEALKEAKKNGYKDIAKAVEKAIKVLEEQRETLRKAREELEKRR
G3BP1	KPGFGVGRGLAPR (13)	453-465	G3bp1-11: MPPRVRRRVEELLRRARELAEELGIRVEVLEVDPEYELELTAVITLAILAVLAPPERQDEVLELLRETLKTLSEVVESLAVSIWAPPEREEAARRVERLVEEAFNTTPETRERARKLRDEARRDAEPDEVVVAVHLVPK
Common gamma chain	ERLCLVSEIP (10)	327-336	IL-2RG-30: SPFREALAAALRELAERHGAHVEVEDYVRVALDPSFGEEERRRRARALARDVAALVAEAAGAEDAEGLAARIEAALLAGERVLFRVVATPEEEAAFLAEAAARGGKLLGVLPLR
Prion	VNITIKQH (8)	180-187	PRI28: MKVTNLTLPVDKSKSVEELKELAVEYFKKMNETGNVEEDLAKLTYYIVQALVLEKEKIEEVLKEIAKELKEAASEKTKVYLEEYIPEMLKEYEETGKVVVKLEVEFEDGTVYEVTGEYIE
BRCA1_ARATH	YTENTVIRLDEHPSLNKEGNL (21)	182-202	BRCA1_ARATH-35: SIKEELLKLIDEAELLDESKKEKIKELASKLSEEAQKKLIELIKEEKEKGNKVVVDYVTPENLEEVLELIEKRVEEDEGKVTFVIFTRYSEKVKEIAGKLKELLEKYPGKVSIILVVAP
FUS	YEPRGRGGGRGGRGGMGGSDRGGFNKFGG (29)	239-267	FUS40: SEFDALLDEMYARGDAARAAGDVEGADLWDTAAIAAEAIAEEDPEKAVEFARKALELLERAAPLSPRERALYVNTELMEFSAKTTGIAVLLVHTALTDEELEELVRRAIELAKKVGGDIILAWSRSARDKAELAERWIVELSK

Supplementary table 1. Summary of IDPs and IDRs and their binders in the study, detailing each protein's sequence and target positional data within their respective structures.

	**Design number**	**Ordered deisgn**	**AF2 interaction pAE**	**RMSD AF2 vs Design**	**AF2 pLDDT for binder**
**Amylin sequence input diffusion**	10,000	96	10	0.5	90
**Amylin partial diffusion**	5000	174	10	0.5	90
**CP sequence input diffusion**	10,000	96	10	0.5	90
**CP partial diffusion**	5000	95	10	0.5	90
**VP48 sequence input diffusion**	30,000	95	16	0.6	87
**VP48-partial diffusion and backbone extension**	25000	95	12	0.3	89
**VP48- 2nd partial diffusion**	8000	48	10	0.3	90
**G3bp1 secondary structrue specification**	10,000	78	10	0.5	90
**G3bp1 partial diffusion**	5,000	95	10	0.5	90
**Prion sequence input diffusion**	7,600	32	6	x	90
**Prion partial diffusion**	20,000	48	6	x	90
**IL2RG secondary structrue specification**	40,000	94	6	x	90
**IL2RG partial diffusion**	20000	46	6.5	x	90
**BRCA1_ARATH sequence input diffusion**	10,000	41	7	1	90
**BRCA1 partial diffusion**	50,000	74	5	1	90
**FUS sequence input diffusion**	30,000	x	15	1	85
**FUS parital diffusion**	20,000	94	9	1	90

Supplementary table 2. Design statistics and AlphaFold2 validation metrics for sequence input and partial diffusion across multiple targets.

Peptide Sequence	Q1 (m/z) (charge state)	Q3 (m/z)	Ion type
KCNTATCATQRLANFLVHSSNNFGAILSST NVGSNTY	976.90 (4+)	921.59	b_{26}^{3+}
		988.48	b_{28}^{3+}
		931.90	b_{36}^{3+}
		541.18	y_5^{+}
GPVGPSGPPGK	475.26 (2+)	795.44	y_9^{+}
		696.37	y_8^{+}
		639.35	y_7^{+}
		398.24	y_4^{+}
		301.19	y_3^{+}
		155.08	b_2^{+}
GPVGPSGPPGK[$^{13}C_6$, $^{15}N_2$]^	479.27 (2+)	704.38	y_8^{+}
		647.36	y_7^{+}
K^ = $^{13}C_6H14^{15}N_2O2$ (+8 Da)		406.25	y_4^{+}

Supplementary Table 3. Amylin transitions monitored.

Mobile phase	Phase A: 0.2% formic acid in water	
	Phase B: 0.2% formic acid in acetonitrile	
Column	Acquity UPLC HSS T3 1.8µm (C18, 2.1x50 mm, pore size 100 Å) (Waters, Milford, MA, P/N 186003539)	
Temperature	45°C	
Flow rate	0.3 mL/min	
Injection volume	10µL	
Gradient	0-0.5 min	20% B at 0.3 mL/min
	7.5 min	60% B at 0.3 mL/min
	9.5 min	98% B at 0.3 mL/min
	11.0 min	98% B at 0.3 mL/min
	11.1 min	20% B at 0.3 mL/min
	12.5 min	20% B at 0.3 mL/min

Supplementary Table 4. Liquid chromatography parameters.

Source Polarity	ESI+
Curtain Gas	35
Collision Gas	9
Ionspray Voltage	5500 V
Source Temperature	400°C
Ion Source Gas 1	40
Ion Source Gas 2	40

Supplementary Table 5. Mass spectrometry parameters.

Supplementary Video

Supplementary Video 1. Sequence input diffusion trajectory for the fully diffused Amylin-binder complex.

This animation illustrates the trajectory generated via sequence input diffusion RFdiffusion with a fully sampled Amylin–binder interface. Beginning from a random distribution of Amylin and binder residues, the trajectory shows how Amylin samples various conformations as the binder organizes around it, progressively folding into a structure that cradles the target's surface.

Supplementary Equation

$$Percent\ recovery_{buffer} = \frac{Group\ C - Group\ B}{Group\ A}$$

$$Percent\ recovery_{Plasma} = \frac{(Group\ G - Group\ F) - (Group\ E - Group\ D)}{Group\ A}$$

Equation 1. Calculation of percent recovery of each binder-coated bead type in LC-MS/MS analysis.
