# Supplementary material for: Diffusing protein binders to intrinsically disordered proteins
Source: Nature. 2025 Jul 30;644(8077):809–17. doi: 10.1038/s41586-025-09248-9 (PMC12367549; doi:10.1038/s41586-025-09248-9)
Supplement: Supplementary file 2 [file 41586_2025_9248_MOESM2_ESM.pdf]

nature portfolio

Corresponding author(s): David Baker

Last updated by author(s): May 19, 2025

Reporting Summary

Nature Portfolio wishes to improve the reproducibility of the work that we publish. This form provides structure for consistency and transparency in reporting. For further information on Nature Portfolio policies, see our Editorial Policies and the Editorial Policy Checklist.

Please do not complete any field with "not applicable" or n/a. Refer to the help text for what text to use if an item is not relevant to your study.
For final submission: please carefully check your responses for accuracy; you will not be able to make changes later.

Statistics

For all statistical analyses, confirm that the following items are present in the figure legend, table legend, main text, or Methods section.

n/a	Confirmed	
☐	☒	The exact sample size (n) for each experimental group/condition, given as a discrete number and unit of measurement
☐	☒	A statement on whether measurements were taken from distinct samples or whether the same sample was measured repeatedly
☒	☐	The statistical test(s) used AND whether they are one- or two-sided<br>*Only common tests should be described solely by name; describe more complex techniques in the Methods section.*
☒	☐	A description of all covariates tested
☒	☐	A description of any assumptions or corrections, such as tests of normality and adjustment for multiple comparisons
☐	☒	A full description of the statistical parameters including central tendency (e.g. means) or other basic estimates (e.g. regression coefficient) AND variation (e.g. standard deviation) or associated estimates of uncertainty (e.g. confidence intervals)
☒	☐	For null hypothesis testing, the test statistic (e.g. F, t, r) with confidence intervals, effect sizes, degrees of freedom and P value noted<br>*Give P values as exact values whenever suitable.*
☐	☒	For Bayesian analysis, information on the choice of priors and Markov chain Monte Carlo settings
☒	☐	For hierarchical and complex designs, identification of the appropriate level for tests and full reporting of outcomes
☒	☐	Estimates of effect sizes (e.g. Cohen's d, Pearson's r), indicating how they were calculated

Our web collection on statistics for biologists contains articles on many of the points above.

Software and code

Policy information about availability of computer code

Data collection	RFdiffusion v1.1.0 (https://github.com/RosettaCommons/RFdiffusion), proteinMPNN V1.0.1 (https://github.com/dauparas/ProteinMPNN), AlphaFold2, NIS Elements 5.30 software. Custom RFdiffusion implementation and inference commands are available via Code Ocean capsule (Capsule title: Diffusing protein binders to intrinsically disordered proteins; available at https://doi.org/10.24433/CO.0868770.v1).
Data analysis	GraphPad Prism version 8, Phenix - 1.21.1_5286, Coot - 0.9.8.7, Molprobity - 4.5.2, Phaser - 2.5.0, PyMOL 2.4.0, UCSF Chimera 1.14, Skyline Daily 23.1.1.459, SoftWoRx 7.0.0, ImageJ 1.54p, FlowJo v9

For manuscripts utilizing custom algorithms or software that are central to the research but not yet described in published literature, software must be made available to editors and reviewers. We strongly encourage code deposition in a community repository (e.g. GitHub). See the Nature Portfolio guidelines for submitting code & software for further information.

Data

Policy information about availability of data

All manuscripts must include a data availability statement. This statement should provide the following information, where applicable:

- Accession codes, unique identifiers, or web links for publicly available datasets
- A description of any restrictions on data availability
- For clinical datasets or third party data, please ensure that the statement adheres to our policy

All data are available in the main text or as supplementary materials. Crystal structures of Amylin-18αβ, Amylin-22αβL and G3bp1-11 have been deposited in Protein Data Bank, with the accession IDs 9NZH, 9CC5 and 9CC6, respectively.

Research involving human participants, their data, or biological material

Policy information about studies with human participants or human data. See also policy information about sex, gender (identity/presentation), and sexual orientation and race, ethnicity and racism.

Reporting on sex and gender	Not applicable
Reporting on race, ethnicity, or other socially relevant groupings	Not applicable
Population characteristics	Not applicable
Recruitment	Not applicable
Ethics oversight	Not applicable

Note that full information on the approval of the study protocol must also be provided in the manuscript.

Field-specific reporting

Please select the one below that is the best fit for your research. If you are not sure, read the appropriate sections before making your selection.

☒ Life sciences ☐ Behavioural & social sciences ☐ Ecological, evolutionary & environmental sciences

For a reference copy of the document with all sections, see nature.com/documents/nr-reporting-summary-flat.pdf

Life sciences study design

All studies must disclose on these points even when the disclosure is negative.

Sample size	No statistical methods were used to pre-determine sample size. Sample sizes were based on prior studies using similar assays and are consistent with field standards. For each experiment, the number of biological replicates is reported in the corresponding figure legend and is sufficient to ensure reproducibility and statistical robustness.
Data exclusions	None
Replication	Each dataset contains many (n reported in figure legends) independent measurements
Randomization	Randomization was not applicable because the study did not involve treatment groups or subject allocation; all samples were processed under identical conditions.
Blinding	Blinding was not applicable as all data acquisition and analysis were performed using standardized protocols and automated software without subjective interpretation.

Reporting for specific materials, systems and methods

We require information from authors about some types of materials, experimental systems and methods used in many studies. Here, indicate whether each material, system or method listed is relevant to your study. If you are not sure if a list item applies to your research, read the appropriate section before selecting a response.

Materials & experimental systems

n/a	Involved in the study
☐	☒ Antibodies
☐	☒ Eukaryotic cell lines
☒	☐ Palaeontology and archaeology
☒	☐ Animals and other organisms
☒	☐ Clinical data
☒	☐ Dual use research of concern
☒	☐ Plants

Methods

n/a	Involved in the study
☒	☐ ChIP-seq
☐	☒ Flow cytometry
☒	☐ MRI-based neuroimaging

Antibodies

Antibodies used: Goat anti-Mouse IgG (H+L) Cross-Adsorbed Secondary Antibody, Alexa Fluor™ 488 (Thermo Fisher Catalog # A-11001); LAMP1 Monoclonal Antibody (H4A3), Alexa Fluor™ 488 (Thermo Fisher, Catalog # MA5-18121)

Validation: Goat anti-Mouse IgG (H+L), Alexa Fluor™ 488 (Thermo Fisher, A-11001) is a secondary antibody validated by the manufacturer for immunofluorescence. It targets mouse IgG heavy and light chains. LAMP1 Monoclonal Antibody (H4A3), Alexa Fluor™ 488 (Thermo Fisher, MA5-18121) is validated by the manufacturer for flow cytometry and immunofluorescence. It reacts with human and mouse LAMP1 and shows expected lysosomal localization.

Eukaryotic cell lines

Policy information about cell lines and Sex and Gender in Research

Cell line source(s): HeLa cells, HEP3B cells

Authentication: The cell lines were not authenticated further after purchase.

Mycoplasma contamination: Cell lines have not recently been tested for mycoplasma contamination

Commonly misidentified lines (See ICLAC register): No commonly misidentified lines were used

Plants

Seed stocks: We didn't use plants

Novel plant genotypes: We didn't use plants

Authentication: We didn't use plants

Flow Cytometry

Plots

Confirm that:

☒ The axis labels state the marker and fluorochrome used (e.g. CD4-FITC).

☒ The axis scales are clearly visible. Include numbers along axes only for bottom left plot of group (a 'group' is an analysis of identical markers).

☒ All plots are contour plots with outliers or pseudocolor plots.

☒ A numerical value for number of cells or percentage (with statistics) is provided.

Methodology

Sample preparation: The cells were first incubated with corresponding protein reagents for indicated time at 37 °C, then washed with cold PBS

Sample preparation	three times. The cells were then stained with corresponding fluorescence-labeled antibodies against the corresponding receptor for 1 hour at room temperature. After washing three times with cold PBS for flow cytometry, flow cytometry was performed in Attune NxT flow cytometer (Thermo Fisher). The data was analyzed in FlowJo software.
Instrument	Sony SH800 / Thermo Attune NxT
Software	Sony SH800S software / Sony Attune NxT software / FlowJo v9
Cell population abundance	For mammalian cell internalization / surface binding assay, at least 2,000 mammalian cells were collected and resuspended in flow focusing buffer per sample.
Gating strategy	For mammalian cell internalization / surface binding assay, the cells were gated based on the major population based on SSC and FSC.

☒ Tick this box to confirm that a figure exemplifying the gating strategy is provided in the Supplementary Information.

This checklist template is licensed under a Creative Commons Attribution 4.0 International License, which permits use, sharing, adaptation, distribution and reproduction in any medium or format, as long as you give appropriate credit to the original author(s) and the source, provide a link to the Creative Commons license, and indicate if changes were made. The images or other third party material in this article are included in the article's Creative Commons license, unless indicated otherwise in a credit line to the material. If material is not included in the article's Creative Commons license and your intended use is not permitted by statutory regulation or exceeds the permitted use, you will need to obtain permission directly from the copyright holder. To view a copy of this license, visit http://creativecommons.org/licenses/by/4.0/
